# Supplementary material for: Safeguarding adolescent mental health in India (SAMA): study protocol for codesign and feasibility study of a school systems intervention targeting adolescent anxiety and depression in India
Source: BMJ Open. 2022 Apr 3;12(4):e054897. doi: 10.1136/bmjopen-2021-054897 (PMC8981280; doi:10.1136/bmjopen-2021-054897)
Supplement: Author's manuscript [file bmjopen-2021-054897.draft_revisions.pdf]

BMJ Open

BMJ Open is committed to open peer review. As part of this commitment we make the peer review history of every article we publish publicly available.

When an article is published we post the peer reviewers' comments and the authors' responses online. We also post the versions of the paper that were used during peer review. These are the versions that the peer review comments apply to.

The versions of the paper that follow are the versions that were submitted during the peer review process. They are not the versions of record or the final published versions. They should not be cited or distributed as the published version of this manuscript.

BMJ Open is an open access journal and the full, final, typeset and author-corrected version of record of the manuscript is available on our site with no access controls, subscription charges or pay-per-view fees (http://bmjopen.bmj.com).

If you have any questions on BMJ Open's open peer review process please email info.bmjopen@bmj.com

BMJ Open

Safeguarding Adolescent Mental Health in India (SAMA): study protocol for co-design and feasibility study of a school systems intervention targeting adolescent anxiety and depression in India

Journal:	*BMJ Open*
Manuscript ID	bmjopen-2021-054897
Article Type:	Protocol
Date Submitted by the Author:	29-Jun-2021

Complete List of Authors:	Hugh-Jones, Siobhan ; University of Leeds Faculty of Biological Sciences, Psychology<br>Janardhana, N; NIMHANS Bengaluru, Department of Psychiatric Social Work; National Institute of Mental Health and Neuro Sciences, Department of Psychiatric Social Work<br>Al-Janabi, Hareth; University of Birmingham, Health Economics Unit, School of Health and Population Sciences<br>Bhola, Poornima; National Institute of Mental Health and Neurosciences, Department of Psychiatric Social Work<br>Cooke, Paul ; University of Leeds Faculty of Arts Humanities and Cultures, School of Languages, Cultures and Societies<br>Fazel, Mina; University of Oxford, Warneford Hospital,, Psychiatry; University of Oxford<br>Hudson, Kristian; Bradford Institute for Health Research, Improvement Academy<br>Khandeparkar, Prachi; Sangath<br>Mirzoev, Tolib; London School of Hygiene & Tropical Medicine, Global Health and Development<br>Venkataraman, Surendran; Indira Gandhi Medical College and Research Institute<br>West, Robert; University of Leeds, Leeds Institute of Health Sciences<br>Mallikarjun, Pavan; University of Birmingham

Keywords:	MENTAL HEALTH, Child & adolescent psychiatry < PSYCHIATRY, PUBLIC HEALTH

SCHOLARONE™ Manuscripts

Safeguarding Adolescent Mental Health in India (SAMA): study protocol for co-design and feasibility study of a school systems intervention targeting adolescent anxiety and depression in India.

Hugh-Jones, Siobhan[a*]., Naidu, Janardhan[b]., Al-Janabi, Hareth[c]., Bhola, Poornima [b]., Cook, Paul[a]., Fazel, Mina[d]., Hudson, Kristian[e]., Khandeparkar, Prachi[f]., Mirzoev, Tolib[g]., Venkataraman, Surendran[h]., West, Robert[a]., Mallikarjun, Pavan.[c]

[a] University of Leeds, Leeds, UK

[b] National Institute of Mental Health and Neurosciences, Bangalore, India

[c] University of Birmingham, Birmingham, UK

[d] University of Oxford, Oxford, UK

[e] Bradford Institute of Health Research, Bradford, UK

[f] Sangath, Goa, India

[g] London School of Hygiene and Tropical Medicine, London, UK

[h] Indira Gandhi Medical College and Research Institute, Pondicherry, India

*Corresponding author: Dr Siobhan Hugh-Jones, School of Psychology, University of Leeds, LS2 9JT. E: S.hugh-jones@leeds.ac.uk. Tel: 0113 343 5744 Fax: 0113 343 549

Keywords: adolescent, anxiety, depression, mental health, schools

Word count: 4418

For peer review only - http://bmjopen.bmj.com/site/about/guidelines.xhtml

Abstract

Introduction

Symptoms of anxiety and depression in Indian adolescents are common. Schools can be opportune sites for delivery of mental health interventions. India, however, is without a whole-school mental health approach. This article describes the study design for the Safeguarding Adolescent Mental Health in India (SAMA) project. The aim of SAMA is to co-design and feasibility test a suite of multi-component interventions across the intersecting systems of adolescents, schools, families and their local communities in India.

Methods and analysis

Our project will co-design and feasibility test four interventions to run in parallel in eight schools (three assigned to waitlist) in Bengaluru and Kolar in Karnataka, India. The primary aim is to reduce the prevalence of adolescent anxiety and depression. Co-design of interventions will build on existing evidence and resources. Interventions for adolescents at school will be universal, incorporating curriculum and social components. Interventions for parents and teachers will target mental health literacy, and also for teachers, training in positive behaviour practices. Intervention in the school community will target school climate to improve student mental health literacy and care. Intervention for the wider community will be via adolescent-led films and social media. We will generate intervention cost estimates, test outcome measures and identify pathways to increase policy action on the evidence.

Ethics and dissemination

Ethical approval has been granted by the National Institute of Mental Health Neurosciences Research Ethics Committee (NIMHANS/26th IEC (Behv Sc Div / 2020/2021)) and the University of Leeds School of Psychology Research Ethics Committee (PSYC-221). Certain data will be available on a data sharing site. Findings will be disseminated via peer-reviewed journals and conferences.

For peer review only - http://bmjopen.bmj.com/site/about/guidelines.xhtml

Article Summary

Strengths and Limitations

1. A key strength of this study is its focus on depression and anxiety prevention, both of which are the common adolescent mental health problems in India.
2. The study utilises a public mental health systems approach, which adopts a 'person-in-context' perspective and recognises multiple determinants of mental health.
3. Extensive co-design of interventions will promote cultural relevance and acceptability by key stakeholders, including adolescents, teachers, parents and school communities.
4. The Covid-19 pandemic makes this research more pertinent but may affect recruitment of the schools / adolescents or may lead to participation bias if the study processes are conducted online by excluding those with limited access or who lack technical skills.
5. The opt-in approach to consent may lead to participation bias.

For peer review only

For peer review only - http://bmjopen.bmj.com/site/about/guidelines.xhtml

Introduction

Mental health conditions including depression and anxiety disorders are among the top ten causes of illness and disability in adolescents.[1] Half of all lifelong mental-health conditions have their onset in adolescence. [2] Poor mental health in adolescence is associated with poor physical health and lifelong disadvantage, especially if education is disrupted, impacting occupational and social trajectories.[3]

India has the largest adolescent population in the world (253 million).[4] An estimated 9.8 million Indian 13–17-year-olds (pooled prevalence of 7.3%) have a clinical mental-health condition [5] but levels seem considerably higher in urban areas and among school-going adolescents. [6] Although there is limited data on the treatment gap in adolescents, the overall treatment gap for mental disorders in India is around 90%. [5] The treatment gap and the effectiveness and cost-effectiveness of early intervention are driving investment into public mental health in efforts to develop scalable programmes that can reach adolescents before their symptoms progress to levels of a clinical disorder.[5] Tackling preventable and treatable health conditions, and improving the quality of education for adolescents, present the single best investments for health and wellbeing a low-and-middle income (LMIC) country can make. [7]

Schools have tremendous potential as platforms for public mental-health interventions, which can secure positive effects on mental health, including a reduction in anxiety and depression, and which can be delivered, sustained and scaled in LMICs. [7] School provision can increase access to interventions over and above existing mental healthcare systems, which in LMICs, are typically fragmented, under-resourced, and not tailored to adolescents. [8–10] Improving school experience may also promote longer participation in education, which reduces a number of risks for girls in particular. [8] The Lancet commission on adolescent wellbeing calls for investment in school mental health, bolstered by the rise in school attendance [8] including in India (97% enrolment). [11] India, however, is without an evidence-based, whole school mental health approach for adolescents.

Our project responds to this need by identifying existing school interventions which can be integrated, tailored and culturally adapted into a systems approach [12] in India through a process of co-production with stakeholders. Given the multiple determinants of adolescent mental health in India, [5] interventions will need to target individual and contextual factors. In India, a key contextual determinant of adolescent mental health is the nature of schooling, including the use of corporal punishment [13–15], extreme academic pressure [16,17], a lack of mental health literacy among adolescents, teachers and parents, [18–20] prevailing mental health stigma and bullying,[21] poorly supported staff, often with large class sizes and a lack

For peer review only - http://bmjopen.bmj.com/site/about/guidelines.xhtml

of mental health support within schools.[22] Although the Government of India endorsed the WHO Health Promoting School model (2007) [23] progress on school mental health has been slow. To date, school initiatives in India have mostly targeted physical health and life skills. [24–27] There is an urgent need to accelerate improvements in mental health in Indian schools, from a safety and rights perspective [28], to reduce school risks to adolescent mental health and to improve population health. India has limited evidence-based, scalable school programmes, endorsed by policy, to support adolescent mental health. This is a critical care gap. [5,29] Our study aims to contribute to the evidence base about what works in Indian schools to support adolescent mental health.

There is some evidence for school-based universal mental interventions for depression and anxiety prevention, and mental health promotion, in High-Income Countries (HICs) [30,31]. The evidence in LMICs is promising though limited [8–10]. Three whole-school health and life-skills programmes with small mental health components have been tested in India. [24,26,32,33] One did not report any beneficial clinical outcomes [24] and one has yet to establish effectiveness. [32] The third is a health promotion programme targeting physical and sexual health, bullying, school climate, gender equality and depressive symptoms. [26] Outcomes were positive, including for depressive symptoms, with benefits extended up to two years [33]. It did not, however, target anxiety nor include broad mental health promotion. This is a critical gap given the prevalence of anxiety disorders in adolescence and its co-morbidity with depression.

Our study **S**afeguarding **A**dolescent **M**ental he**A**lth in India (SAMA) will co-design, and feasibility test, a suite of school interventions in India targeting multiple risks and protective factors across the school system. The health objective of these interventions is to reduce symptoms of anxiety and depression in symptomatic adolescents and to promote the wellbeing all school-going adolescents. The SAMA study will take place between January 2021 and December 2023.

For peer review only

For peer review only - http://bmjopen.bmj.com/site/about/guidelines.xhtml

Method and analysis

Study design

SAMA will run over 36 months and will deliver 8 work packages (WP) (see Figure 1).

WP1-4 are intervention co-design / co-adaptation and feasibility studies following MRC guidance [34] and the Six Steps in Quality Intervention Development 6SQuID [35], targeting adolescents (14–15 years), teachers, the school community and parents (Table 1). WP5 is implementation research on these interventions and WP6 is an exploratory cost-effectiveness study of the interventions. WP7 and 8 target policy and local community systems respectively. Year 1 will focus on co-design and co-adaptation of interventions via WP1-5. Years 2 and 3 will include a feasibility study of the adapted interventions alongside WP6 and 7. WP8 will run across all three years.

SAMA will work with adolescents as partners in all stages of the project. In addition to the adolescents involved as participants in co-design, we will have a Youth Advisory Board who will be consulted on all project components, a subset of whom will form adolescent film crews and drive the social media campaigning (both WP8). We will have three other advisory boards (Steering Group, Scientific Advisory Board, Ethics Oversight Committee).

----Figure 1: Study work packages----

For WP1-4, some broad interventions requirements have been defined in advance of co-design, including the aims and primary and secondary outcomes. These are shown in Table 1. All interventions will be delivered by external lay counsellors. There is some evidence that this delivery approach is superior and preferred to teacher-delivered interventions, and peer educator programmes, at least for adolescent mental health programmes. [26] [22] [32] There are additional benefits of having a small team of lay counsellors working closely in partnership with schools.

For peer review only - http://bmjopen.bmj.com/site/about/guidelines.xhtml

Table 1: Intervention aims and requirements defined in advance

WP	Intervention	Requirements defined prior to co-design	Primary outcome	Secondary outcomes (include but not restricted to)
1	Whole school curriculum adolescent mental health programme	15-16y (Grade 9) as advised by schools<br><br>6-8 weeks duration	Symptoms of anxiety and depression	Mental health literacy, stress, self-esteem, resilience, quality of life, school attendance and performance.
2	Teacher education programme	Low intensity<br><br>Suitable for all levels of staff	Teacher mental health literacy	Teacher attitude towards and use of positive behaviour management practices.
3	School climate intervention	Up to 6 months	School climate	Mental health literacy, perceived stigma, perceived support
4	Parent mental health literacy intervention	Low intensity	Parent mental health literacy	Requests for more mental health information / support / referrals

School recruitment

We will recruit eight schools in Kolar and Bangalore to participate in both the co-design and feasibility stages of SAMA (WP1–6). School recruitment will be facilitated by the Karnataka state primary and secondary education ministry and the Karnataka state secondary school Headmaster's Association. We will recruit two school types (government and low-cost private) to identify school factors that may affect feasibility. Schools will be eligible to take part in SAMA if they endorse the delivery of WP1–6 in their schools, including participation in co-design. WP1 intervention feasibility testing will be with a new Grade 9 cohort not involved in co-design.

Year 1 Co-design / co-adaptation of interventions

Co-design will proceed in stages (see Figure 2) and will include co-adaptation of existing interventions as well as design of new components where needed (e.g. content, implementation, safeguarding). We begin with co-adapting interventions before identifying the need for new co-designed elements. Our approach to co-adaptation is informed by phases 1–3 of the ADAPT model for introducing complex public health interventions into new settings [36] and also by Perera et al.'s approach [37] to adapting low-intensity psychological interventions into new cultural settings. WP5 runs alongside WP1–4 and aims to identify implementation facilitators and barriers for all intervention components. As well as adapting interventions, we will co-produce with adolescent and stakeholders a whole-school safeguarding protocol tailored to the interventions.

For peer review only - http://bmjopen.bmj.com/site/about/guidelines.xhtml

----Figure 2: Stages of co-adaptation / co-design---

Phase 1: Exploration of needs and existing interventions

Initial assessment

A desk review will be conducted to gather knowledge on the local context and research evidence relevant to our project aims, including identification of any existing best practice and policy requirements. This will feed into an evidence-based, multi-level needs assessment of end-users, families, schools and regional / state systems to sensitise our selection of interventions and implementation approach.

Intervention selection & exploration

We aim to identify interventions that are likely to be acceptable, feasible and efficacious in our target context to meet our project aims. We will search for interventions with the potential (after co-adaptation) to impact at least the primary outcome for each WP (Table 1). As per Figure 2, a non-systematic search will be conducted first to collate or identify interventions which are known to the team in India, referenced in Indian education or mental health policies or referenced by Indian or global organisations (e.g. Save the Children, WHO). This will be followed by a systematic search. Umbrella review protocols for these are registered on Prospero [38–41] and reviews are nearing completion. Considering search returns from both the non-systematic and systematic searched, WP1–4 leads will propose candidate interventions to be taken to Phase 2. Whilst established effectiveness of an intervention is important, we also need to consider the coherence, integration, implementation and scalability of interventions for the local context. [42] Proposals will be reviewed by the research team and project advisory boards, which include in-country adolescents, schools and mental health professionals as well as the team which led SEHER [26]. Amendments to proposals will be made where necessary.

This phase includes work on implementation protocols (WP5). We will build on a UK framework for a whole-school mental health intervention [43] based on the Consolidated Framework for Implementation Research (CFIR) [44]. The framework will be iteratively developed as SAMA interventions are finalised, drawing on existing implementation evidence from India, including the SEHER study [26], and through our co-adaptation processes.

For peer review only - http://bmjopen.bmj.com/site/about/guidelines.xhtml

Phase 2: Preparation for co-design

Identification of mismatches and model development

Candidate interventions will be assessed against our multi-level needs assessment; those failing to meet significant need will be rejected. We will then develop a model of how the final interventions could work across systems before identifying the aspects (across surface and deep levels [45]) which are most critical to take forward to co-design / co-adaptation. Decisions here will be informed by the approach of Perera et al. [37] and their use of the Ecological Validity Model [46]. This will lead to the generation of adaptation hypotheses, that is, our proposals for which intervention aspects will need to be culturally adapted. The Ecological Validity Model prompts directs consideration of the language, persons, metaphors, content, concepts, goals, methods and context (which includes implementation) of the intervention to be adapted. We will supplement these proposals with co-design needs, that is, new features which need to be created because of the systems approach or local school context. Co-adaptation and co-design proposals will be finalised in consultation with our youth and advisory boards.

Co-adaptation and co-design

Our aim in this stage is to optimise the acceptability, feasibility, efficacy and meaningful evaluation of the suite of interventions by involving end-users and stakeholders in intervention adaptation and its associated implementation approach. We will host co-design / co-adaptation workshops for each WP intervention, each involving adolescents (14–16y, single gendered groups with more females), parents, schools (including teachers and institute heads) and key stakeholders in separate workshops. WP2 co-design will additionally involve national organisations (e.g. State Education & Health Department, Teachers of India, ChildLine India, and Save the Children). WP4 co-design will begin with a stakeholder event to identify community-led solutions to reaching parents. Creative evaluation methods will be devised during co-design to be sensitive to low literacy levels, low chance of measure completion and desirability bias. Implementation barriers, facilitators and developing implementation protocol will be a standing item for consultation in co-design workshops. Co-design / co-adaptation workshops will include a range of age- and role-appropriate discussions and activities centering on the co-adaptation proposals developed in the previous stage.

For peer review only - http://bmjopen.bmj.com/site/about/guidelines.xhtml

Phase 3: Undertaking Modifications

We will modify the interventions based on co-adaptation and co-design workshop outputs. We will utilise the expanded FRAME approach to report modifications to interventions. [47] This approach documents when, why and how modifications were made, and differentiates cultural adaptation from adaptations made for other reasons. Proposed modifications will be reviewed by the full team to ensure modified interventions still meet the multi-level needs assessment, retain active ingredients and are coherent as a suite of interventions. Modifications and final prototype interventions will be reviewed by our youth and advisory boards as well as a board of in-country mental health professionals and education sector representatives to ensure they meet state and national requirements. Once finalised, we will produce for each WP intervention a logic model, programme manual, intervention resources, lay counsellor training and supervision protocol, implementation and evaluation protocols.

Year 2 Feasibility testing

Lay counsellors will receive full training prior to the launch of the feasibility study. The feasibility study will run in eight schools, with three randomly assigned to waitlist. The four interventions will run in parallel in schools. Lay counsellors will complete weekly monitoring logs of intervention delivery in each school, including logging of modifications made (as per FRAME [47] and will have weekly supervision by clinical team members. Fidelity monitoring will be undertaken by a team of trained data collection assistants.

We have established preliminary feasibility indicators for each intervention, which will be updated when interventions are finalised. Feasibility indicators which will be common across WP1–4 interventions are: meeting recruitment and retention rates, delivery as intended, measure completion and no significant adverse effects. We will adopt a ‘traffic-light’ system for recruitment and retention as follows: Red – if rates below 50% (say) then will not proceed; Amber – if rates 50-65% then proceed if there are plans to improve; Green – above 65% proceed to definitive trial. Mixed-methods process and evaluation data will be secured for all interventions from adolescents, teachers, parents and lay counsellors to explore: perceived usefulness of the interventions; implementation challenges; unintended negative effects; safeguarding challenges; the process of change and intervention development needs. Evaluation outcomes will inform intervention improvements and parameters for a definitive trial.

For peer review only - http://bmjopen.bmj.com/site/about/guidelines.xhtml

Sample size calculation

The planned sample for the feasibility study by intervention is detailed in Table 2. Calculation of power is based on WP1 and on an analysis of variance approach for simplicity. We will test two candidate primary outcomes measures for WP1, the Strengths and Difficulties Questionnaire (SDQ) [48] and the Revised Child Anxiety and Depression Scale (RACDS) [49]. SDQ is an emotional and behavioural screening tool, culturally validated in India and used widely in adolescent mental health research. RCADS measures anxiety and depression and demonstrates excellent sensitivity-to-change. A translated RCADS has been used with Indian populations [50] but full cultural validity needs to be established. As this will be an important tool to strengthen Indian mental health research, we will conduct a full validation study in parallel to the co-design stage. We have determined via a UK school feasibility trial [51] that the change in SDQ had a standard deviation of 2.8 points. Assuming 80% recruitment, comparing the mean changes in SDQ from 480 participants from the intervention group with 288 from the waitlist group using a 1% significance level has more than 98% power to detect a difference of 1 point on the SDQ scale. Around 960 adolescents will be approached for WP1. At 65% recruitment, the power remains over 95%.

Table 2: Planned sample size for each intervention in the feasibility study

WP	Intervention	N	Sample
1	Universal curriculum adolescent mental health programme	960	Grade 9 15–16-year-olds from 8 schools (120 per school)
2	Teacher education programme	32	Grade 9 teachers (4 per school)
3	School climate intervention	8 schools	To be defined following intervention selection
4	Parental mental literacy intervention	960 sets of parents	Aim to reach all parents / carers of WP1 participants

WP5 Implementation research

We will test the feasibility of our co-designed implementation approach, which will draw on improvement science. Lay counsellors will be trained and supported to optimise the intervention in situ by logging and overcoming implementation challenges as they arise, whilst keeping fidelity to intervention function. Process evaluations based on interviews / focus groups and implementation assessment tools will be conducted post-intervention with key school staff and other stakeholder as needed depending on what challenges arise. Solutions to implementation challenges will be sought.

For peer review only - http://bmjopen.bmj.com/site/about/guidelines.xhtml

WP6 Health economic evaluation

Using a micro (bottom-up) costing strategy [52], separate design and delivery costs per intervention component will be estimated using available data sources (e.g. government ministry data, national pay scales, national earnings surveys) to determine unit costs for personnel time. Any additional primary data needs will be identified. Costs will be presented at the class and school level to promote generalisability. Preference-based outcome health and wellbeing measures are needed for economic evaluation [53] but there is limited evidence on their use with adolescents and no evidence of their use feasibility in this context (with adolescents in school-based settings and in India). We will feasibility test candidate measures including CHU-9D [54] and EQ-5D-Y [45] via pre-post administration to 50 intervention adolescents and via 10 think-aloud interviews to identify completion challenges. We will also administer the EQ-5D-5L [56] and ICECAP-A [57] pre- and post-intervention to 50 parents to determine measure completion and any need for instrument adaptation. We will use the cost and outcome data to generate exploratory cost-consequence analyses of the intervention strategies.

WP7 Understanding barriers and opportunities for research to policy uptake

We aim to (i) understand facilitators, barriers and solutions to policy uptake of the evidence on school mental health (in Karnataka); (ii) identify ways of increasing adolescents' participation in policy processes, and (ii) identify the potential for application of solutions to other states. A policy and stakeholder mapping exercise will be undertaken, and then key advisors (n~10) for each of the three policies (two National and one state) [29] will be purposefully identified and interviewed. Insights will be shared at a stakeholder event comprising about 20 education and health decision-makers, adolescents and teachers to generate solutions for policy improvement. Insights will direct a social media campaign for policy action. Evaluation of effectiveness will be based on the number and nature of stakeholders reached, new knowledge on evidence-to-policy barriers and solutions and adolescents' participation.

WP8 Community Film making and social media campaign.

Around 15 adolescents (single-gendered groups and more females) will be supported to create 3 short films on experiences and risks around adolescent mental health, the potential for the school to affect mental health, and solutions to safeguard their well-being. Films will

For peer review only - http://bmjopen.bmj.com/site/about/guidelines.xhtml

be used in WP1-4 for engagement, education, advocacy and impact, and to inform (i) a community and stakeholder's event. Films will be used in WP1-4 for engagement, education, advocacy and impact, via (i) a community and stakeholder's event to screen the films to promote shared understanding and solutions; (ii) a social media education campaign targeting adolescents' mental health literacy; and (iii) an advocacy campaign targeting national-level policymakers. Outcomes will include open access films and new knowledge on the feasibility and reach of community filmmaking for health education and advocacy campaigning for school mental health in India.

Data analysis plan

WP1-5 Co-design / co-adaptation

Workshop data will include audio / video recordings, facilitator notes and design materials (e.g. maps, user journeys, reference ranking, annotated implementation and safeguarding protocols). Key workshop moments which informed an adaptation or new design component will be transcribed, supplemented with facilitator notes and accuracy checked by two facilitators. Workshop data from different stakeholder groups per focus (e.g. intervention, implementation, safeguarding) will be integrated by WP leads. They will synthesise proposed modifications and report them as per FRAME [47] to take forward to final approval stages in preparation for the feasibility study (described above).

WP1–5 Feasibility Study

School-level effects will be handled as fixed rather than random effects. Primary analysis will be analysis of covariance where outcomes at three months are regressed upon the baseline value and any relevant covariates/factors as age, sex, school, participant characteristics. The analysis will be supported by a two-level mixed-effects model where measures at two-time points are nested within participants. If further to this analysis, a model is required to examine the relationship between the change in measure over time and the initial measure level, then Bloomqvist's method will be employed. Process evaluations will be analysed using Framework Analysis [58] or Thematic Analysis [59]. The implementation achievements in each school will be quantified per intervention and as a systems approach, and actions associated with success will be identified, drawing on the CFIR [44] and as per Hudson et al. [43].

For peer review only - http://bmjopen.bmj.com/site/about/guidelines.xhtml

WP6 Health economic evaluation

For the quantitative validity testing of the outcomes measures, we will calculate completion rates of the measures and items. For the qualitative validity testing. we will code the think-aloud interview transcripts to identify errors or difficulties in responding to items. Mean pre- and post-quality of life scores will be used to estimate quality of life benefits from the interventions. These data will be combined with estimates of the intervention costs in a simple cost–consequence analysis [60].

WP7 Understanding barriers and opportunities for research to policy uptake

Data from the stakeholder analysis and document reviews will be continuously triangulated with analysis of transcripts from the in-depth interviews. All qualitative data will be analysed using Framework Approach [58] and guided by the three questions for WP7 and relevant theoretical frameworks for evidence-informed policymaking. [61-64]

Patient and public involvement

Throughout SAMA, listening to adolescents will be central and incorporated in each WP. There are many barriers to adolescent involvement in low resource settings.[31] These can include high levels of mental health stigma as well as cultural differences where adolescent's opinions might be less commonly sought and heard. Our approach to adolescent participation will be informed by the WHO Global Consensus Statement on Meaningful Adolescent & Youth Engagement. [65] Adolescents will be trained and supported to contribute to the project. As well as being involved as participants in the design and evaluation of interventions in WP1-5, other adolescents will sit our Youth Advisory Panel to act as 'critical friends' of the project. Adolescent contribution will also be via participatory filmmaking. This is a key platform within and beyond our project for youth voice in India on adolescent mental health and the role of schools in wellbeing. Other stakeholders including parents, teachers, the school community and policymakers will be involved in the co-design and evaluation stages of the study.

Ethics and dissemination

This research is being conducted in community settings and has received ethical approval from the National Institute of Mental Health Neurosciences Research Ethics Committee (NIMHANS/26th IEC (Behv Sc Div / 2020/2021)), and the University of Leeds School of Psychology Research Ethics Committee (PSYC-221). Additional approvals have been

For peer review only - http://bmjopen.bmj.com/site/about/guidelines.xhtml

granted by the Karnataka state primary and secondary education ministry and the Karnataka Department of State Education Research and Training.

We will seek consent from parents, teachers, school staff and other stakeholders and assent from adolescents. Grade 9 pupils will receive WP1 and 3 but can opt out of data collection. This is to ensure that participants' rights are not undermined by universal delivery. Teachers can access WP2 training without consenting to data collection. This is in recognition of the sensitivity of teachers reporting their mental health knowledge or use of harsh discipline practices, and reponses will inform our understanding of the acceptability of our data collection measures. Audio/video recording in workshops and for WP8 will require explicit consent. Research data will be kept secure and confidential, with agreed UK–India data management plans and data sharing agreements.

Alongside WP1–5, we will be drafting, and refining through co-design, a stepped response and referral protocol to be assessed as part of the feasibility study. This protocol will draw on national and international guidance, as well as established practice in NIMHANS. Our priorities are response protocols for identification of (i) moderate to serious mental health need or risk (especially suicide risk) and (ii) abuse (especially sexual abuse). The iterative production and field testing of this school mental health safeguarding policy will be an important contribution to the research field as well as to school systems.

Our plans for dissemination include: (i) events – launch, film screening, impact and capacity building events; (ii) website – project website with multiple functionalities to engage, share resources, disseminate findings and in ways that can be sustained beyond the project; (iii) social media (twitter, Instagram and TikTok) to reach multiple sectors and adolescents; (iv) project briefs/updates/newsletters (flyers), especially for schools and community organisations; (v) executive reports to key stakeholders (e.g. education and health departments, teacher training colleges, mental health service providers); (vi) sharing the project on global repositories, such as the Mental Health Innovation Network; (vii) using existing communication platforms from University of Leeds Global Challenges project relevant to adolescent wellbeing in LMICs; and (viii) conferences and peer-reviewed publications.

Authors contributions: All authors contributed to the design of the protocol and approved the final version. SHJ and PM produced early manuscript drafts and all other authors reviewed and edited the manuscript.

For peer review only - http://bmjopen.bmj.com/site/about/guidelines.xhtml

Funding statement

This work is supported by the UK's Medical Research Council, Economic and Social Research Council, National Institute of Health Research and UK Aid (grant number MR/T040238/1). All views expressed here are of the authors only.

Competing interests statement.

The authors declare no competing interests.

References

1 Vos T, Lim SS, Abbafati C, *et al.* Global burden of 369 diseases and injuries in 204 countries and territories, 1990–2019: a systematic analysis for the Global Burden of Disease Study 2019. *Lancet* 2020;**396**:1204–22. doi:10.1016/S0140-6736(20)30925-9

2 Kessler RC, Angermeyer M, Anthony JC, *et al.* Lifetime prevalence and age-of-onset distributions of mental disorders in the World Health Organization's World Mental Health Survey Initiative. *World Psychiatry* 2007;**6**:168–76. Pmid:https://pubmed.ncbi.nlm.nih.gov/18188442/

3 Patton GC, Coffey C, Romaniuk H, *et al.* The prognosis of common mental disorders in adolescents: a 14-year prospective cohort study. *Lancet* 2014;**383**:1404–11. doi:10.1016/S0140-6736(13)62116-9

4 Adolescent Development and Participation, Available: https://www.unicef.org/india/what-we-do/adolescent-development-participation [Accessed 25 June 2021]

5 Gautham MS, Gururaj G, Varghese M, *et al*. The National Mental Health Survey of India (2016): Prevalence, socio-demographic correlates and treatment gap of mental morbidity. *Int J Soc Psychiatry* 2020; **66**(4): 361-372. doi.org/10.1177/0020764020907941

6 Aggarwal S, Berk M. Evolution of adolescent mental health in a rapidly changing socioeconomic environment: a review of mental health studies in adolescents in India over last 10 years. *Asian J Psychiatr* 2015;**13**:3–12. doi:10.1016/j.ajp.2014.11.007

7 Patton GC, Sawyer SM, Santelli JS, *et al.* Our future: a Lancet commission on adolescent health and wellbeing. *Lancet* 2016;**387**:2423–78. doi:10.1016/S0140-6736(16)00579-1

8 Barry MM, Clarke AM, Jenkins R, *et al.* A systematic review of the effectiveness of mental health promotion interventions for young people in low and middle-income countries. *BMC Public Health* 2013;**13**:835. doi:10.1186/1471-2458-13-835

9 Bradshaw M, Gericke H, Coetzee BJ, *et al.* Universal school-based mental health programmes in low- and middle-income countries: A systematic review and narrative synthesis. *Prev Med* 2021;**143**:106317. doi:10.1016/j.ypmed.2020.106317

10 Fazel M, Patel V, Thomas S, *et al.* Mental health interventions in schools in low-income and middle-income countries. *Lancet Psychiatry* 2014;**1**:388–98. doi:10.1016/S2215-0366(14)70357-8

11 Annual State Education Report. ASER 2018 Full report. New Delhi: ASER Centre 2019.

For peer review only - http://bmjopen.bmj.com/site/about/guidelines.xhtml

12 Bronfenbrenner U. Ecological systems theory. In: *Six theories of child development: Revised formulations and current issues*. Jessica Kingsley Publishers, 1992:187–249.

13 Deb S, Kumar A, Holden GW, *et al.* School corporal punishment, family tension, and students' internalizing problems: Evidence from India. *School Psychol Int* 2017;**38**:60–77. doi:10.1177/0143034316681378

14 Jyoti S. Implications of corporal punishment on primary school children. *IOSR J Hum Soc Sci* 2013;**15**:57–61. doi:10.9790/0837-1565761

15 Corporal Punishment in Schools Longitudinal Evidence from Ethiopia, India Peru and Viet Nam, 2015. Available: https://www.unicef-irc.org/publications/pdf/CORPORAL%20PUNISHMENT%20IDP2finalrev.pdf

16 Arun P, Garg R, Chavan BS. Stress and suicidal ideation among adolescents having academic difficulty. *Ind Psychiatry J* 2017;**26**:64–70. doi:10.4103/ipj.ipj_5_17

17 Deb S, Strodl E, Sun J. Academic Stress, Parental Pressure, Anxiety and Mental Health among Indian High School Students. *Int J Psychol Behav Sci* 2015;**5**:26–34. 10.5923/j.ijpbs.20150501.04

18 Venkataraman S, Patil R, Balasundaram S. Stigma toward mental illness among higher secondary school teachers in Puducherry, South India. *J Family Med Prim Care* 2019;**8**:1401–7. doi:10.4103/jfmpc.jfmpc_203_19

19 Parikh N, Parikh M, Vankar G, *et al.* Knowledge and attitudes of secondary and higher secondary school teachers toward mental illness in Ahmedabad. *Indian J Soc Psychiatry*;**32**:56. doi:10.4103/0971-9962.176770

20 Venkataraman S, Patil R, Balasundaram S. Why mental health literacy still matters: a review. *Int J Community Med Public Health* 2019;**6**:2723–9. doi:10.18203/2394-6040.ijcmph20192350

21 Kumar A. Cultures of learning in developing education systems: Government and NGO classrooms in India. *Int J of Educ Res* 2019;**95**:76–89. doi:10.1016/j.ijer.2019.02.009

22 Parikh R, Michelson D, Sapru M, *et al.* Priorities and preferences for school-based mental health services in India: a multi-stakeholder study with adolescents, parents, school staff, and mental health providers. *Glob Ment Health (Camb)* 2019;**6**. doi:10.1017/gmh.2019.16

23 Health promoting schools. https://www.who.int/health-topics/health-promoting-schools#tab=tab_1 [(accessed 25 June 2021]).

24 Srikala B, Kishore KKV. Empowering adolescents with life skills education in schools – School mental health program: Does it work? *Indian J Psychiatry* 2010;**52**:344–9. doi:10.4103/0019-5545.74310

25 Kumar D, Bharath S, Hirisave U, *et al.* School mental health programs in India: Current status and future directions. In: Weist MD, Kutcher S, Wei Y, eds. *School Mental Health: Global Challenges and Opportunities*. Cambridge University Press 2015: 95–104. doi:10.1017/CBO9781107284241.009

26 Shinde S, Weiss HA, Varghese B, *et al.* Promoting school climate and health outcomes with the SEHER multi-component secondary school intervention in Bihar, India: a cluster-

For peer review only - http://bmjopen.bmj.com/site/about/guidelines.xhtml

randomised controlled trial. *Lancet* 2018;**392**:2465–77. doi:10.1016/S0140-6736(18)31615-5

27 Gujar K, Pingale V. School mental health programme in Maharashtra - Today and way ahead. *Ann Indian Psychiatry*;**1**:4. doi:10.4103/aip.aip_14_17

28 The State of the World's Children 2011: Adolescence - An age of opportunity. UNICEF DATA, 2011. Available: https://data.unicef.org/resources/the-state-of-the-worlds-children-2011-adolescents-an-age-of-opportunity/ [Accessed 8 May 2021].

29 Roy K, Shinde S, Sarkar BK, *et al.* India's response to adolescent mental health: a policy review and stakeholder analysis. *Soc Psychiatr Epidemiol* 2019;**54**:405–14. doi:10.1007/s00127-018-1647-2

30 Werner-Seidler A, Perry Y, Calear AL, *et al.* School-based depression and anxiety prevention programs for young people: A systematic review and meta-analysis. *Clin Psychol Rev* 2017;**51**:30–47. doi:10.1016/j.cpr.2016.10.005

31 Fazel M, Hoagwood K, Stephan S, *et al.* Mental health interventions in schools 1. *Lancet Psychiatry* 2014;**1**:377–87. doi:10.1016/S2215-0366(14)70312-8

32 Rajaraman D, Travasso S, Chatterjee A, *et al.* The acceptability, feasibility and impact of a lay health counsellor delivered health promoting schools programme in India: a case study evaluation. *BMC Health Serv Res* 2012;**12**:127. doi:10.1186/1472-6963-12-127

33 Shinde S, Weiss HA, Khandeparkar P, *et al.* A multicomponent secondary school health promotion intervention and adolescent health: An extension of the SEHER cluster randomised controlled trial in Bihar, India. *PLoS Med* 2020;**17**:e1003021. doi:10.1371/journal.pmed.1003021

34 Craig P, Dieppe P, Macintyre S, *et al.* Developing and evaluating complex interventions: the new Medical Research Council guidance. *BMJ* 2008;**337**:a1655. doi:10.1136/bmj.a1655

35 Wight D, Wimbush E, Jepson R, *et al.* Six steps in quality intervention development (6SQuID). *J Epidemiol Community Health* 2016;**70**:520–5. doi:10.1136/jech-2015-205952

36 Movsisyan, A., Arnold, L., Evans, R. et al. Adapting evidence-informed complex population health interventions for new contexts: a systematic review of guidance. *Implement Sci* 2019; **14**:105. https://doi.org/10.1186/s13012-019-0956-5

37 Perera, C., Salamanca-Sanabria, A., Caballero-Bernal, J. et al. No implementation without cultural adaptation: a process for culturally adapting low-intensity psychological interventions in humanitarian settings. *Confl Health* 2020;**14**: 46. https://doi.org/10.1186/s13031-020-00290-0

38 Hugh-Jones S, Prabhu S, Lakshman KA, *et al.* The effectiveness of interventions to improve secondary school teachers' mental health literacy: a protocol for an umbrella review CRD42021250458. Published Online First: 2021. http://www.crd.york.ac.uk/PROSPERO/display_record.asp?ID=CRD42021250458

39 Hugh-Jones S, Prabhu S, Warriner L, *et al.* The effectiveness of universal school interventions for prevention or treatment of adolescent anxiety and depression: a

For peer review only - http://bmjopen.bmj.com/site/about/guidelines.xhtml

protocol for an umbrella review. CRD42021248592. Published Online First: 2021. http://www.crd.york.ac.uk/PROSPERO/display_record.asp?ID=CRD42021248592

40 Hugh-Jones S, Prabhu S, Podiya JK, *et al.* The effectiveness of interventions to improve school climate in secondary schools: a protocol for an umbrella review. CRD42021251790. Published Online First: 2021. http://www.crd.york.ac.uk/PROSPERO/display_record.asp?ID=CRD42021251790

41 Hugh-Jones S, Prabhu S, Nag R, *et al.* The effectiveness of interventions to improve parent mental health literacy: a protocol for an umbrella review. CRD42021252006. Published Online First: 2021. http://www.crd.york.ac.uk/PROSPERO/display_record.asp?ID=CRD42021252006

42 Boustani M, Daleiden E, Bernstein A, *et al.* Using relevance mapping methodology to design an adolescent mental health intervention in India. *Glob Health Action* 2020; **13**:1. doi: 10.1080/16549716.2020.1775062

43 Hudson KG, Lawton R, Hugh-Jones S. (2020). Factors affecting the implementation of a whole school mindfulness program: a qualitative study using the consolidated framework for implementation research. *BMC Health Serv Res* 2020;**20**: 1-13. https://doi.org/10.1186/s12913-020-4942-z

44 Damschroder LJ, Aron DC, Keith RE, *et al.* Fostering implementation of health services research findings into practice: a consolidated framework for advancing implementation science. *Implement Sci* 2009;**4**:50. doi:10.1186/1748-5908-4-50

45 Stirman, S.W., Miller, C.J., Toder, K. et al. Development of a framework and coding system for modifications and adaptations of evidence-based interventions. *Implement Sci* 2013; **8**: 65). https://doi.org/10.1186/1748-5908-8-65

46 Bernal G, Sáez-Santiago E. Culturally centered psychosocial interventions. *J Community Psychol* 2006;**34:**121–32. doi:https://doi.org/10.1002/jcop.20096

47 Stirman, SW, Baumann AA, Miller, CJ. The FRAME: an expanded framework for reporting adaptations and modifications to evidence-based interventions. *Implement Sci2019; **14***(1), 1-10.

48 Goodman R. Psychometric Properties of the Strengths and Difficulties Questionnaire. *J Am Acad Child Adolesc Psychiatry* 2001;**40**:1337–45. doi:10.1097/00004583-200111000-00015

49 Chorpita BF, Moffitt CE, Gray J. Psychometric properties of the Revised Child Anxiety and Depression Scale in a clinical sample. *Behav Res Ther* 2005;**43**:309–22. doi:10.1016/j.brat.2004.02.004

50 Wolpert M, Cheng H, Deighton J. Measurement Issues: Review of four patient reported outcome measures: SDQ, RCADS, C/ORS and GBO - their strengths and limitations for clinical use and service evaluation. *Child Adolesc Ment Health* 2015;**20**:63–70. doi:10.1111/camh.12065

51 Hugh-Jones S, Pert K, Kendal S, *et al.* MindMate2U: Development and feasibility study of a co-designed school-based mHealth intervention for symptomatic adolescents. JMIR Preprints. 22/04/2021:29834

For peer review only - http://bmjopen.bmj.com/site/about/guidelines.xhtml

52 Berry V, Axford N, Blower S, *et al.* The Effectiveness and Micro-costing Analysis of a Universal, School-Based, Social–Emotional Learning Programme in the UK: A Cluster-Randomised Controlled Trial. *School Ment Health* 2016;**8**:238–56. doi:10.1007/s12310-015-9160-1

53 Brazier J, Ratcliffe J, Saloman J, *et al. Measuring and Valuing Health Benefits for Economic Evaluation*. Oxford University Press 2017.

54 Stevens K. Developing a descriptive system for a new preference-based measure of health-related quality of life for children. *Qual Life Res* 2009;**18**:1105–13. doi:10.1007/s11136-009-9524-9

55 Ravens-Sieberer U, Wille N, Badia X, *et al.* Feasibility, reliability, and validity of the EQ-5D-Y: results from a multinational study. *Qual Life Res* 2010;**19**:887–97. doi:10.1007/s11136-010-9649-x

56 Herdman M, Gudex C, Lloyd A, *et al.* Development and preliminary testing of the new five-level version of EQ-5D (EQ-5D-5L). *Qual Life Res* 2011;**20**:1727–36. doi:10.1007/s11136-011-9903-x

57 Al-Janabi H, Flynn TN, Coast J. Development of a self-report measure of capability wellbeing for adults: the ICECAP-A. *Qual Life Res* 2012;**21**:167–76. doi:10.1007/s11136-011-9927-2

58 Gale NK, Heath G, Cameron E, *et al*. Using the framework method for the analysis of qualitative data in multi-disciplinary health research. BMC Med Res Methodol 2013; **13**: 117 (2013). https://doi.org/10.1186/1471-2288-13-117

59 Terry G, Hayfield N, Clarke V, *et al.* Thematic analysis. *The Sage Handbook of Qualitative Research in Psychology* 2017; pp 17-37.

60 Coast J. Is economic evaluation in touch with society's health values? *BMJ* 2004;**329**:1233–6. doi:10.1136/bmj.329.7476.1233

61 Mirzoev T, Das M, Ebenso B, *et al.* Contextual influences on the role of evidence in health policy development: what can we learn from six policies in India and Nigeria? *Evid Policy 2017;* **13**(1):59-79.

62 Mirzoev T, Green A, Gerein N, *et al.* 2013. Role of evidence in maternal health policy processes in Vietnam, India and China: findings from the HEPVIC project. *Evid Policy* 2013; **9**(4):493-511.

63 Lavis J, Oxman A, Lewin S, *et al.* SUPPORT Tools for evidence-informed health Policymaking (STP). *Health Res Policy Syst 2009;*7(Suppl 1):I1.

64 Green A, Gadsby E. 2007. A framework for evidence-informed health policy-making. In: *Green A, Bennett S, editors. Sound Choices: Enhancing Capacity for Evidence-Informed Health Policy AHPSR Biennual Review*. WHO, Alliance for Health Policy and Systems Research.

65 Global Consensus Statement, Meaningful Adolescent & Youth Engagement, 2018. Available: https://www.who.int/pmnch/mye-statement.pdf?ua=1 [Accessed 25 June 2021].

For peer review only - http://bmjopen.bmj.com/site/about/guidelines.xhtml

For peer review only

For peer review only - http://bmjopen.bmj.com/site/about/guidelines.xhtml

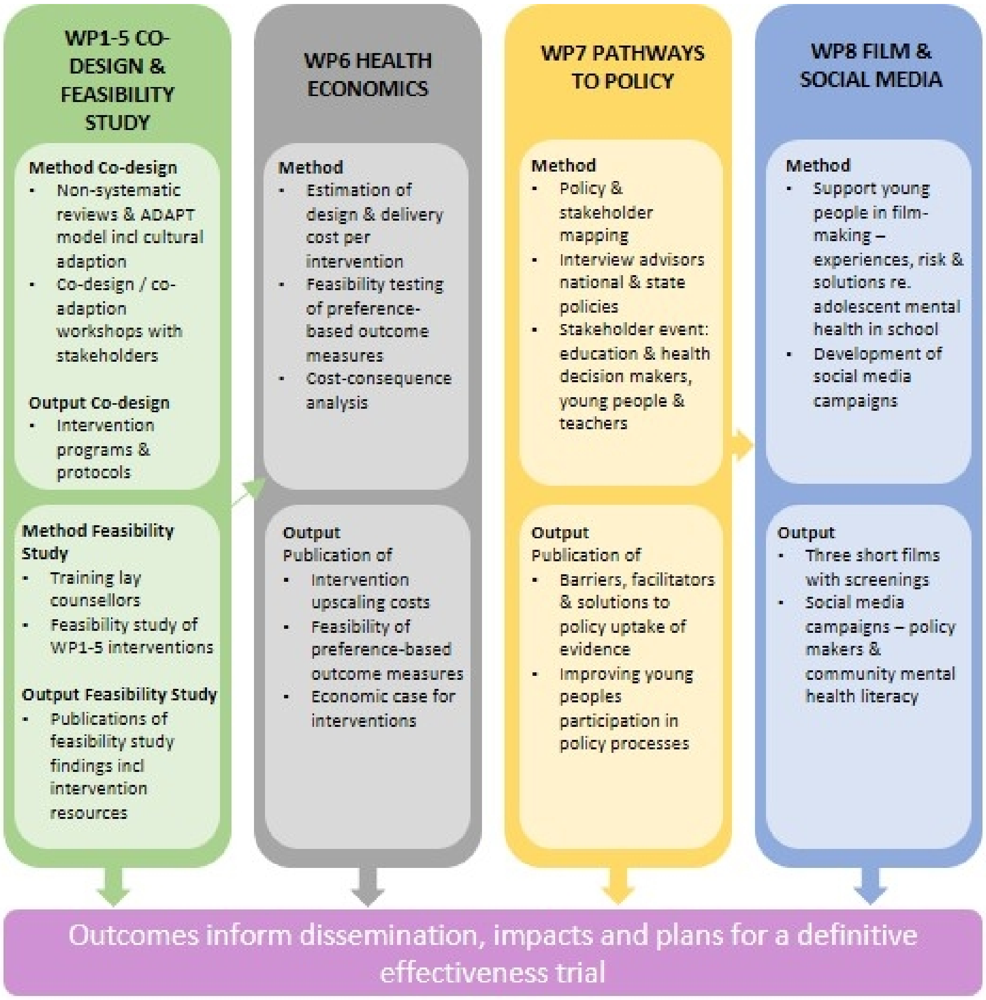

Fig 1 Study work packages

89x90mm (150 x 150 DPI)

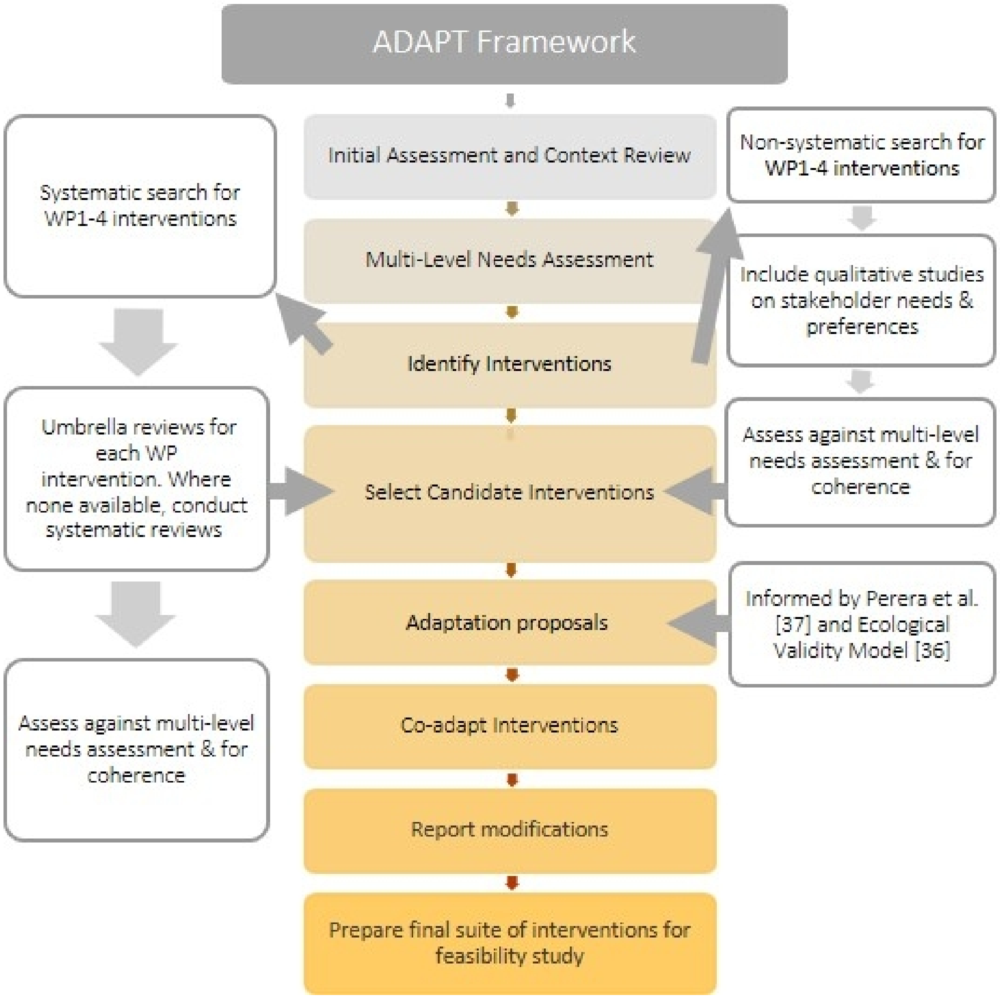

Figure 2 Stages of co-adaptation

90x90mm (150 x 150 DPI)

Safeguarding Adolescent Mental Health in India (SAMA): study protocol for co-design and feasibility study of a school systems intervention targeting adolescent anxiety and depression in India

Journal:	*BMJ Open*
Manuscript ID	bmjopen-2021-054897.R1
Article Type:	Protocol
Date Submitted by the Author:	01-Dec-2021
Complete List of Authors:	Hugh-Jones, Siobhan ; University of Leeds Faculty of Biological Sciences, Psychology<br>Janardhana, N; NIMHANS Bengaluru, Department of Psychiatric Social Work; National Institute of Mental Health and Neuro Sciences, Department of Psychiatric Social Work<br>Al-Janabi, Hareth; University of Birmingham, Health Economics Unit, School of Health and Population Sciences<br>Bhola, Poornima; National Institute of Mental Health and Neurosciences, Department of Psychiatric Social Work<br>Cooke, Paul ; University of Leeds Faculty of Arts Humanities and Cultures, School of Languages, Cultures and Societies<br>Fazel, Mina; University of Oxford, Warneford Hospital,, Psychiatry; University of Oxford<br>Hudson, Kristian; Bradford Institute for Health Research, Improvement Academy<br>Khandeparkar, Prachi; Sangath<br>Mirzoev, Tolib; London School of Hygiene & Tropical Medicine, Global Health and Development<br>Venkataraman, Surendran; Indira Gandhi Medical College and Research Institute<br>West, Robert; University of Leeds, Leeds Institute of Health Sciences<br>Mallikarjun, Pavan; University of Birmingham
**Primary Subject Heading**:	Mental health
Secondary Subject Heading:	Public health, Global health
Keywords:	MENTAL HEALTH, PUBLIC HEALTH, PREVENTIVE MEDICINE

Safeguarding Adolescent Mental Health in India (SAMA): study protocol for co-design and feasibility study of a school systems intervention targeting adolescent anxiety and depression in India.

Hugh-Jones, Siobhan[a*]., Naidu, Janardhan[b]., Al-Janabi, Hareth[c]., Bhola, Poornima [b]., Cook, Paul[a]., Fazel, Mina[d]., Hudson, Kristian[e]., Khandeparkar, Prachi[f]., Mirzoev, Tolib[g]., Venkataraman, Surendran[h]., West, Robert[a]., Mallikarjun, Pavan.[c]

[a] University of Leeds, Leeds, UK

[b] National Institute of Mental Health and Neurosciences, Bangalore, India

[c] University of Birmingham, Birmingham, UK

[d] University of Oxford, Oxford, UK

[e] Bradford Institute of Health Research, Bradford, UK

[f] Sangath, Goa, India

[g] London School of Hygiene and Tropical Medicine, London, UK

[h] Indira Gandhi Medical College and Research Institute, Pondicherry, India

*Corresponding author: Dr Siobhan Hugh-Jones, School of Psychology, University of Leeds, LS2 9JT. E: S.hugh-jones@leeds.ac.uk. Tel: 0113 343 5744 Fax: 0113 343 549

Keywords: adolescent, anxiety, depression, mental health, schools

Word count: 4848

For peer review only

Abstract

Introduction

Symptoms of anxiety and depression in Indian adolescents are common. Schools can be opportune sites for delivery of mental health interventions. India, however, is without a whole-school mental health approach. This article describes the study design for the Safeguarding Adolescent Mental Health in India (SAMA) project. The aim of SAMA is to co-design and feasibility test a suite of multi-component interventions across the intersecting systems of adolescents, schools, families and their local communities in India.

Methods and analysis

Our project will co-design and feasibility test four interventions to run in parallel in eight schools (three assigned to waitlist) in Bengaluru and Kolar in Karnataka, India. The primary aim is to reduce the prevalence of adolescent anxiety and depression. Co-design of interventions will build on existing evidence and resources. Interventions for adolescents at school will be universal, incorporating curriculum and social components. Interventions for parents and teachers will target mental health literacy, and also for teachers, training in positive behaviour practices. Intervention in the school community will target school climate to improve student mental health literacy and care. Intervention for the wider community will be via adolescent-led films and social media. We will generate intervention cost estimates, test outcome measures and identify pathways to increase policy action on the evidence.

Ethics and dissemination

Ethical approval has been granted by the National Institute of Mental Health Neurosciences Research Ethics Committee (NIMHANS/26th IEC (Behv Sc Div / 2020/2021)) and the University of Leeds School of Psychology Research Ethics Committee (PSYC-221). Certain data will be available on a data sharing site. Findings will be disseminated via peer-reviewed journals and conferences.

For peer review only

Article Summary

Strengths and Limitations

1. A key strength of this study is its focus on depression and anxiety prevention, both of which are the common adolescent mental health problems in India.
2. The study utilises a public mental health systems approach, which adopts a 'person-in-context' perspective and recognises multiple determinants of mental health.
3. Extensive co-design of interventions will promote cultural relevance and acceptability by key stakeholders, including adolescents, teachers, parents and school communities.
4. The Covid-19 pandemic makes this research more pertinent but may affect recruitment of the schools / adolescents or may lead to participation bias if the study processes are conducted online by excluding those with limited access or who lack technical skills.
5. The opt-in approach to consent may lead to participation bias.

For peer review only

For peer review only - http://bmjopen.bmj.com/site/about/guidelines.xhtml

Introduction

Mental health conditions including depression and anxiety disorders are among the top ten causes of illness and disability in adolescents.[1] Half of all lifelong mental-health conditions have their onset in adolescence. [2] Poor mental health in adolescence is associated with poor physical health and lifelong disadvantage, especially if education is disrupted, impacting occupational and social trajectories.[3]

India has the largest adolescent population in the world (253 million).[4] An estimated 9.8 million Indian 13–17-year-olds (pooled prevalence of 7.3%) have a clinical mental-health condition [5] but levels seem considerably higher in urban areas and among school-going adolescents. [6] Although there is limited data on the treatment gap in adolescents, the overall treatment gap for mental disorders in India is around 90%. [5] The treatment gap and the effectiveness and cost-effectiveness of early intervention are driving investment into public mental health in efforts to develop scalable programmes that can reach adolescents before their symptoms progress to levels of a clinical disorder.[5] Tackling preventable and treatable health conditions, and improving the quality of education for adolescents, present the single best investments for health and wellbeing a low-and-middle income (LMIC) country can make. [7]

Schools have tremendous potential as platforms for public mental-health interventions, which can secure positive effects on mental health, including a reduction in anxiety and depression, and which can be delivered, sustained and scaled in LMICs. [7] School provision can increase access to interventions over and above existing mental healthcare systems, which in LMICs, are typically fragmented, under-resourced, and not tailored to adolescents. [8–10] Improving school experience may also promote longer participation in education, which reduces a number of risks for girls in particular. [8] The Lancet commission on adolescent wellbeing calls for investment in school mental health, bolstered by the rise in school attendance [8] including in India (97% enrolment). [11] India, however, is without an evidence-based, whole school mental health approach for adolescents.

Our project responds to this need by identifying existing school interventions which can be integrated, tailored and culturally adapted into a systems approach [12] in India through a process of co-production with stakeholders. Given the multiple determinants of adolescent mental health in India, [5] interventions will need to target individual and contextual factors. In India, a key contextual determinant of adolescent mental health is the nature of schooling, including the use of corporal punishment [13–15], extreme academic pressure [16,17], a lack of mental health literacy among adolescents, teachers and parents, [18–20] prevailing mental health stigma and bullying,[21] poorly supported staff, often with large class sizes and a lack

For peer review only - http://bmjopen.bmj.com/site/about/guidelines.xhtml

of mental health support within schools.[22] Although the Government of India endorsed the WHO Health Promoting School model (2007) [23] progress on school mental health has been slow. To date, school initiatives in India have mostly targeted physical health and life skills. [24–27] There is an urgent need to accelerate improvements in mental health in Indian schools, from a safety and rights perspective [28], to reduce school risks to adolescent mental health and to improve population health. India has limited evidence-based, scalable school programmes, endorsed by policy, to support adolescent mental health. This is a critical care gap. [5,29] Our study aims to contribute to the evidence base about what works in Indian schools to support adolescent mental health.

There is some evidence for school-based universal mental interventions for depression and anxiety prevention, and mental health promotion, in High-Income Countries (HICs) [30,31]. The evidence in LMICs is promising though limited [8–10]. Three whole-school health and life-skills programmes with small mental health components have been tested in India. [24,26,32,33] One did not report any beneficial clinical outcomes [24] and one has yet to establish effectiveness. [32] indicating that life-skills interventions may be insufficient to impact adolescent anxiety and depression. The third is a health promotion programme (SEHER) focusing on school climate, and targeting physical and sexual health, bullying, gender equality and depressive symptoms. It focused on promoting adolescents' social and problem-solving skills, engaging adolescents, teachers, and parents in school-level decision-making and delivering factual knowledge about health and risk behaviours to the school community. [26] Outcomes were positive, including for depressive symptoms, with benefits extended up to two years [33]. It did not, however, target anxiety nor include broad mental health promotion. This is a critical gap given the prevalence of anxiety disorders in adolescence and its co-morbidity with depression.

Our study **S**afeguarding **A**dolescent **M**ental he**A**lth in India (SAMA) builds on learning from SEHER in terms of the value and acceptability of whole school, multi-component interventions in India schools and the contribution of school climate to adolescent well-being. Project SAMA extends SEHER in several ways. Our health objective is to reduce symptoms of both anxiety and depression in symptomatic adolescents and to promote the wellbeing all school-going adolescents. We will co-design, and feasibility test, a suite of school interventions in India targeting multiple risks and protective factors across the school system. We extend SEHER by including co-designed interventions targeting teachers and parents, as well as an adolescent mental health psychoeducation intervention. We also augment the school climate intervention of SEHER via co-production. The SAMA study will take place between January 2021 and December 2023.

For peer review only - http://bmjopen.bmj.com/site/about/guidelines.xhtml

Method and analysis

Study design

SAMA will run over 36 months and will deliver 8 work packages (WP) (see Figure 1).

WP1-4 are intervention co-design / co-adaptation and feasibility studies following MRC guidance [34] and the Six Steps in Quality Intervention Development 6SQuID [35], targeting adolescents (14–15 years), teachers, the school community and parents (Table 1). WP5 is implementation research on these interventions and WP6 is an exploratory cost-effectiveness study of the interventions. WP7 and 8 target policy and local community systems respectively. Year 1 will focus on co-design and co-adaptation of interventions via WP1-5. Years 2 and 3 will include a feasibility study of the adapted interventions alongside WP6 and 7. WP8 will run across all three years.

SAMA will work with adolescents as partners in all stages of the project. In addition to the adolescents involved as participants in co-design, we will have a Youth Advisory Board who will be consulted on all project components, a subset of whom will form adolescent film crews and drive the social media campaigning (both WP8). We will have three other advisory boards (Steering Group, Scientific Advisory Board, Ethics Oversight Committee).

----Figure 1: Study work packages----

For WP1-4, some broad interventions requirements have been defined in advance of co-design, including the aims and primary and secondary outcomes. These are shown in Table 1. It is possible that co-design discussions may lead to the inclusion of additional outcome measures. All interventions will be delivered by external lay counsellors. Use of lay counsellors can represent effective task-shifting task, an approach endorsed by the World Health Organization in low-resource settings. [36] Task-shifting involves assigning tasks and responsibilities from more specialised to less specialised groups and requires clearly defined roles with effective training and supervision. There is some evidence that this delivery approach is superior and preferred to teacher-delivered interventions, and peer educator programmes, at least for adolescent mental health programmes. [22] [26] [32] Notably, the cluster randomised trial of SEHER (a multicomponent school health promotion program) conducted across 75 schools in Goa was found to be effective when delivered by lay counsellors but not teachers. [26] There are additional benefits of having a small team of lay counsellors working closely in partnership with schools. These include each lay counsellors having a co-counsellor to work alongside to deliver SAMA, counsellors being able to get to

For peer review only - http://bmjopen.bmj.com/site/about/guidelines.xhtml

know particular schools well (i.e. their systems, culture), having constancy in the lay counsellors so school staff and students can build familiarity and trust, and having a defined conduit for communication about SAMA within schools and between school and the study team.

Table 1: Intervention aims and requirements defined in advance

WP	Intervention	Requirements defined prior to co-design	Primary outcome	Secondary outcomes (include but not restricted to)
1	Whole school curriculum adolescent mental health programme	15-16y (Grade 9) as advised by schools<br><br>6-8 weeks duration	Symptoms of anxiety and depression	Mental health literacy, stress, self-esteem, resilience, quality of life, school attendance and performance.
2	Teacher education programme	Low intensity<br><br>Suitable for all levels of staff	Teacher mental health literacy	Teacher attitude towards and use of positive behaviour management practices.
3	School climate intervention	Up to 6 months	School climate	Mental health literacy, perceived stigma, perceived support
4	Parent mental health literacy intervention	Low intensity	Parent mental health literacy	Requests for more mental health information / support / referrals

For peer review only

School recruitment

We will recruit eight schools in Kolar and Bangalore to participate in both the co-design and feasibility stages of SAMA (WP1–6). School recruitment will be facilitated by the Karnataka state primary and secondary education ministry and the Karnataka state secondary school Headmaster's Association. We will recruit two school types (government and low-cost private) to identify school factors that may affect feasibility. Schools will be eligible to take part in SAMA if they endorse the delivery of WP1–6 in their schools, including participation in co-design. WP1 intervention feasibility testing will be with a new Grade 9 cohort not involved in co-design.

Year 1 Co-design / co-adaptation of interventions

Co-design will proceed in stages (see Figure 2) and will include co-adaptation of existing interventions as well as design of new components where needed (e.g. content, implementation, safeguarding). We begin with co-adapting interventions before identifying the need for new co-designed elements. Our approach to co-adaptation is informed by

For peer review only - http://bmjopen.bmj.com/site/about/guidelines.xhtml

phases 1–3 of the ADAPT model for introducing complex public health interventions into new settings [37] and also by Perera et al.'s approach [38] to adapting low-intensity psychological interventions into new cultural settings. WP5 runs alongside WP1–4 and aims to identify implementation facilitators and barriers for all intervention components. As well as adapting interventions, we will co-produce with adolescent and stakeholders a whole-school safeguarding protocol tailored to the interventions.

----Figure 2: Stages of co-adaptation / co-design---

Phase 1: Exploration of needs and existing interventions

Initial assessment

A desk review will be conducted to gather knowledge on the local context and research evidence relevant to our project aims, including identification of any existing best practice and policy requirements. This will feed into an evidence-based, multi-level needs assessment of end-users, families, schools and regional / state systems to sensitise our selection of interventions and implementation approach.

Intervention selection & exploration

We aim to identify interventions that are likely to be acceptable, feasible and efficacious in our target context to meet our project aims. We will search for interventions with the potential (after co-adaptation) to impact at least the primary outcome for each WP (Table 1). As per Figure 2, a non-systematic search will be conducted first to collate or identify interventions which are known to the team in India, referenced in Indian education or mental health policies or referenced by Indian or global organisations (e.g. Save the Children, WHO). This will be followed by a systematic search. Umbrella review protocols for these are registered on Prospero [39–42] and reviews are nearing completion. Considering search returns from both the non-systematic and systematic searched, WP1–4 leads will propose candidate interventions to be taken to Phase 2. Whilst established effectiveness of an intervention is important, we also need to consider the coherence, integration, implementation and scalability of interventions for the local context. [43] Proposals will be reviewed by the research team and project advisory boards, which include in-country adolescents, schools and mental health professionals as well as the team which led SEHER [26]. Amendments to proposals will be made where necessary.

For peer review only - http://bmjopen.bmj.com/site/about/guidelines.xhtml

This phase includes work on implementation protocols (WP5). We will build on a UK framework for a whole-school mental health intervention [44] based on the Consolidated Framework for Implementation Research (CFIR) [45]. The framework will be iteratively developed as SAMA interventions are finalised, drawing on existing implementation evidence from India, including the SEHER study [26], and through our co-adaptation processes.

Phase 2: Preparation for co-design

Identification of mismatches and model development

Candidate interventions will be assessed against our multi-level needs assessment; those failing to meet significant need will be rejected. We will then develop a model of how the final interventions could work across systems before identifying the aspects (across surface and deep levels [46]) which are most critical to take forward to co-design / co-adaptation. Decisions here will be informed by the approach of Perera et al. [38] and their use of the Ecological Validity Model [47]. This will lead to the generation of adaptation hypotheses, that is, our proposals for which intervention aspects will need to be culturally adapted. The Ecological Validity Model prompts directs consideration of the language, persons, metaphors, content, concepts, goals, methods and context (which includes implementation) of the intervention to be adapted. We will supplement these proposals with co-design needs, that is, new features which need to be created because of the systems approach or local school context. Co-adaptation and co-design proposals will be finalised in consultation with our youth and advisory boards.

Co-adaptation and co-design

Our aim in this stage is to optimise the acceptability, feasibility, efficacy and meaningful evaluation of the suite of interventions by involving end-users and stakeholders in intervention adaptation and its associated implementation approach. We will host co-design / co-adaptation workshops for each WP intervention, each involving adolescents (14–16y, single gendered groups with more females), parents, schools (including teachers and institute heads) and key stakeholders in separate workshops. WP2 co-design will additionally involve national organisations (e.g. State Education & Health Department, Teachers of India, ChildLine India, and Save the Children). WP4 co-design will begin with a stakeholder event to identify community-led solutions to reaching parents. Creative evaluation methods will be devised during co-design to be sensitive to low literacy levels, low chance of measure completion and desirability bias. Implementation barriers, facilitators and developing implementation protocol will be a standing item for consultation in co-design workshops. Co-

For peer review only - http://bmjopen.bmj.com/site/about/guidelines.xhtml

design / co-adaptation workshops will include a range of age- and role-appropriate discussions and activities centering on the co-adaptation proposals developed in the previous stage.

Phase 3: Undertaking Modifications

We will modify the interventions based on co-adaptation and co-design workshop outputs. We will utilise the expanded FRAME approach to report modifications to interventions. [48] This approach documents when, why and how modifications were made, and differentiates cultural adaptation from adaptations made for other reasons. Proposed modifications will be reviewed by the full team to ensure modified interventions still meet the multi-level needs assessment, retain active ingredients and are coherent as a suite of interventions. Modifications and final prototype interventions will be reviewed by our youth and advisory boards as well as a board of in-country mental health professionals and education sector representatives to ensure they meet state and national requirements. Once finalised, we will produce for each WP intervention a logic model, programme manual, intervention resources, lay counsellor training and supervision protocol, implementation and evaluation protocols.

Year 2 Feasibility testing

Lay counsellors will be from the local areas, have at least a Masters qualification in psychology or a related discipline and experience in working with young people and / or schools. Prior to the launch of the feasibility study, they will receive full training over three months by a clinical psychologist on the team, supported by another team member experienced in training lay counsellors. Trainees will deliver multiple mock sessions of intervention delivery to support readiness for delivery in schools and quality control. The feasibility study will run in eight schools, with three randomly assigned to waitlist. The four interventions will run in parallel in schools. Lay counsellors will complete weekly monitoring logs of intervention delivery in each school, including logging of modifications made (as per FRAME [48]) and will have weekly supervision by clinical team members. Fidelity monitoring will be undertaken by a team of trained data collection assistants who have been heavily involved in the intervention development stage and will be highly familiar with the implementation protocol. Fidelity monitoring reports will be discussed frequently with the research manager to identify needs for lay counsellor action / support or intervention modification (see WP5).

For peer review only - http://bmjopen.bmj.com/site/about/guidelines.xhtml

We have established preliminary feasibility indicators for each intervention, which will be updated when interventions are finalised. Feasibility indicators which will be common across WP1–4 interventions are: meeting recruitment and retention rates, delivery as intended, measure completion and no significant adverse effects. We will adopt a 'traffic-light' system for recruitment and retention as follows: Red – if rates below 50% (say) then will not proceed; Amber – if rates 50-65% then proceed if there are plans to improve; Green – above 65% proceed to definitive trial. Mixed-methods process and evaluation data will be secured for all interventions from adolescents, teachers, parents and lay counsellors to explore: perceived usefulness of the interventions; implementation challenges; unintended negative effects; safeguarding challenges; the process of change and intervention development needs. Evaluation outcomes will inform intervention improvements and parameters for a definitive trial.

Sample size calculation

The planned sample for the feasibility study by intervention is detailed in Table 2. The minimum recommended sample size for a feasibility study is 30. [49] The smallest group of participants are teachers (for the WP2 intervention). To secure a sample of 30, we will need to recruit a minimum of 8 schools, from which we will then be able to recruit approximately n=960 Grade 9 adolescents (for WP1 intervention). Given this adolescent sample size, we have been able to calculate power in relation to WP1, where a key aim is to test two candidate primary outcomes measures - the Strengths and Difficulties Questionnaire (SDQ) [50] and the Revised Child Anxiety and Depression Scale (RACDS) [51]. SDQ is an emotional and behavioural screening tool, culturally validated in India and used widely in adolescent mental health research. RCADS measures anxiety and depression and demonstrates excellent sensitivity-to-change. A translated RCADS has been used with Indian populations [52] but full cultural validity needs to be established. As this will be an important tool to strengthen Indian mental health research, we will conduct a full validation study in parallel to the co-design stage. We have determined via a UK school feasibility trial [53] that the change in SDQ had a standard deviation of 2.8 points. Assuming 80% recruitment, comparing the mean changes in SDQ from 480 participants from the intervention group with 288 from the waitlist group using a 1% significance level has more than 98% power to detect a difference of 1 point on the SDQ scale. Around 960 adolescents will be approached for WP1. At 65% recruitment, the power remains over 95%. This calculation is based on an analysis of variance approach, that is, regressing the change in SDQ upon the intervention status (intervention or waiting list). The power achieved from an

For peer review only - http://bmjopen.bmj.com/site/about/guidelines.xhtml

analysis of covariance approach, which regresses the final SDQ score upon baseline and intervention status, should be similar. Should the mechanism of the WP1 intervention be similar across population subgroups, we can therefore show good power. There may, however, be differences in the operation of the intervention between subgroups, such as government versus private schools. In that case, assuming an even division of samples, the power becomes 85% with 80% recruitment and 78% with 65% recruitment. A significance level of 1% is used rather than 5% since the sensitivity of the outcome measures is sought to be compared – that is, the SDQ will be compared in sensitivity in this setting with the R-CADS.

Table 2: Planned sample size for each intervention in the feasibility study

WP	Intervention	N	Sample
1	Universal curriculum adolescent mental health programme	960	Grade 9 15–16-year-olds from 8 schools (120 per school)
2	Teacher education programme	32	Grade 9 teachers (4 per school)
3	School climate intervention	8 schools	To be defined following intervention selection
4	Parental mental literacy intervention	960 sets of parents	Aim to reach all parents / carers of WP1 participants

WP5 Implementation research

We will test the feasibility of our co-designed implementation approach, which will draw on improvement science. Lay counsellors will be trained and supported to optimise the intervention in situ by logging and overcoming implementation challenges as they arise, whilst keeping fidelity to intervention function. Process evaluations based on interviews / focus groups and implementation assessment tools will be conducted post-intervention with key school staff and other stakeholder as needed depending on what challenges arise. Solutions to implementation challenges will be sought.

WP6 Health economic evaluation

Using a micro (bottom-up) costing strategy [54], separate design and delivery costs per intervention component will be estimated using available data sources (e.g. government ministry data, national pay scales, national earnings surveys) to determine unit costs for personnel time. Any additional primary data needs will be identified. Costs will be presented at the class and school level to promote generalisability. Preference-based outcome health

For peer review only - http://bmjopen.bmj.com/site/about/guidelines.xhtml

and wellbeing measures are needed for economic evaluation [55] but there is limited evidence on their use with adolescents and no evidence of their use feasibility in this context (with adolescents in school-based settings and in India). We will feasibility test candidate measures including CHU-9D [56] and EQ-5D-Y [57] via pre-post administration to 50 intervention adolescents and via 10 think-aloud interviews to identify completion challenges (a satisfactory sample size given the richness of interview data). We will also administer the EQ-5D-5L [58] and ICECAP-A [59] pre- and post-intervention to 50 parents to determine measure completion and any need for instrument adaptation. These sample sizes are not selected to estimate the significance of a specific outcome, but to balance the desire to not over-burden individuals while still informing completion rates of instruments and items. We will use the cost and outcome data to generate exploratory cost-consequence analyses of the intervention strategies.

WP7 Understanding barriers and opportunities for research to policy uptake

We aim to (i) understand facilitators, barriers and solutions to policy uptake of the evidence on school mental health (in Karnataka); (ii) identify ways of increasing adolescents' participation in policy processes, and (ii) identify the potential for application of solutions to other states. A policy and stakeholder mapping exercise will be undertaken, and then key advisors (n~10) for each of the three policies (two National and one state) [29] will be purposefully identified and interviewed. Insights will be shared at a stakeholder event comprising about 20 education and health decision-makers, adolescents and teachers to generate solutions for policy improvement. Insights will direct a social media campaign for policy action. Evaluation of effectiveness will be based on the number and nature of stakeholders reached, new knowledge on evidence-to-policy barriers and solutions and adolescents' participation.

WP8 Community Film making and social media campaign.

Around 15 adolescents (single-gendered groups and more females) will be supported to create 3 short films on experiences and risks around adolescent mental health, the potential for the school to affect mental health, and solutions to safeguard their well-being. Films will be used in WP1-4 for engagement, education, advocacy and impact, and to inform (i) a community and stakeholder's event. Films will be used in WP1-4 for engagement, education, advocacy and impact, via (i) a community and stakeholder's event to screen the films to promote shared understanding and solutions; (ii) a social media education campaign targeting adolescents' mental health literacy; and (iii) an advocacy campaign targeting

For peer review only - http://bmjopen.bmj.com/site/about/guidelines.xhtml

national-level policymakers. Outcomes will include open access films and new knowledge on the feasibility and reach of community filmmaking for health education and advocacy campaigning for school mental health in India.

Data analysis plan

WP1-5 Co-design / co-adaptation

Workshop data will include audio / video recordings, facilitator notes and design materials (e.g. maps, user journeys, reference ranking, annotated implementation and safeguarding protocols). Key workshop moments which informed an adaptation or new design component will be transcribed, supplemented with facilitator notes and accuracy checked by two facilitators. Workshop data from different stakeholder groups per focus (e.g. intervention, implementation, safeguarding) will be integrated by WP leads. They will synthesise proposed modifications and report them as per FRAME [48] to take forward to final approval stages in preparation for the feasibility study (described above).

WP1–5 Feasibility Study

School-level effects will be handled as fixed rather than random effects. Primary analysis will be analysis of covariance where outcomes at three months are regressed upon the baseline value and any relevant covariates/factors as age, sex, school, participant characteristics. The analysis will be supported by a two-level mixed-effects model where measures at two-time points are nested within participants. If further to this analysis, a model is required to examine the relationship between the change in measure over time and the initial measure level, then Bloomqvist's method will be employed. Process evaluations will be analysed using Framework Analysis [60] or Thematic Analysis [61]. The implementation achievements in each school will be quantified per intervention and as a systems approach, and actions associated with success will be identified, drawing on the CFIR [45] and as per Hudson et al. [44].

WP6 Health economic evaluation

For the quantitative validity testing of the outcomes measures, we will calculate completion rates of the measures and items. For the qualitative validity testing. we will code the think-aloud interview transcripts to identify errors or difficulties in responding to items. Mean pre- and post-quality of life scores will be used to estimate quality of life benefits from the

For peer review only - http://bmjopen.bmj.com/site/about/guidelines.xhtml

interventions. These data will be combined with estimates of the intervention costs in a simple cost–consequence analysis [62].

WP7 Understanding barriers and opportunities for research to policy uptake

Data from the stakeholder analysis and document reviews will be continuously triangulated with analysis of transcripts from the in-depth interviews. All qualitative data will be analysed using Framework Approach [58] and guided by the three questions for WP7 and relevant theoretical frameworks for evidence-informed policymaking. [63-66]

For peer review only

Patient and public involvement

Throughout SAMA, listening to adolescents will be central and incorporated in each WP. There are many barriers to adolescent involvement in low resource settings.[31] These can include high levels of mental health stigma as well as cultural differences where adolescent's opinions might be less commonly sought and heard. Our approach to adolescent participation will be informed by the WHO Global Consensus Statement on Meaningful Adolescent & Youth Engagement. [67] Adolescents will be trained and supported to contribute to the project. As well as being involved as participants in the design and evaluation of interventions in WP1-5, other adolescents will sit our Youth Advisory Panel to act as 'critical friends' of the project. Adolescent contribution will also be via participatory filmmaking. This is a key platform within and beyond our project for youth voice in India on adolescent mental health and the role of schools in wellbeing. Other stakeholders including parents, teachers, the school community and policymakers will be involved in the co-design and evaluation stages of the study.

Ethics and dissemination

This research is being conducted in community settings and has received ethical approval from the National Institute of Mental Health Neurosciences Research Ethics Committee (NIMHANS/26th IEC (Behv Sc Div / 2020/2021)), and the University of Leeds School of Psychology Research Ethics Committee (PSYC-221). Additional approvals have been granted by the Karnataka state primary and secondary education ministry and the Karnataka Department of State Education Research and Training.

We will seek consent from parents, teachers, school staff and other stakeholders and assent from adolescents. Grade 9 pupils will receive WP1 and 3 but can opt out of data collection. This is to ensure that participants' rights are not undermined by universal delivery. Teachers

For peer review only - http://bmjopen.bmj.com/site/about/guidelines.xhtml

can access WP2 training without consenting to data collection. This is in recognition of the sensitivity of teachers reporting their mental health knowledge or use of harsh discipline practices, and responses will inform our understanding of the acceptability of our data collection measures. Audio/video recording in workshops and for WP8 will require explicit consent. Research data will be kept secure and confidential, with agreed UK–India data management plans and data sharing agreements.

Alongside WP1–5, we will be drafting, and refining through co-design, a stepped response and referral protocol to be assessed as part of the feasibility study. This protocol will draw on national and international guidance, as well as established practice in NIMHANS. Our priorities are response protocols for identification of (i) moderate to serious mental health need or risk (especially suicide risk) and (ii) abuse (especially sexual abuse). The iterative production and field testing of this school mental health safeguarding policy will be an important contribution to the research field as well as to school systems.

Our plans for dissemination include: (i) events – launch, film screening, impact and capacity building events; (ii) website – project website with multiple functionalities to engage, share resources, disseminate findings and in ways that can be sustained beyond the project; (iii) social media (twitter, Instagram ~~and TikTok~~) to reach multiple sectors and adolescents; (iv) project briefs/updates/newsletters (flyers), especially for schools and community organisations; (v) executive reports to key stakeholders (e.g. education and health departments, teacher training colleges, mental health service providers); (vi) sharing the project on global repositories, such as the Mental Health Innovation Network; (vii) using existing communication platforms from University of Leeds Global Challenges project relevant to adolescent wellbeing in LMICs; and (viii) conferences and peer-reviewed publications.

Contributorship statement: SHJ is guarantor. SHJ, JN, PM, PB, MF, HA, PC, RW and TM designed the study. PM and SHJ prepared the first draft of the manuscript with contribution from HA on health economics, from TM on policy pathways and PC on filmmaking. RW assisted with writing the feasibility study design and data analysis; KH assisted with writing about implementation science; MF, PB and PK assisted with writing about PPIE, lay counsellors, ethics and safeguarding; and SV assisted with writing about mental health literacy. All authors reviewed and approved the final draft.

Funding statement

This work is supported by the UK's Medical Research Council, Economic and Social Research Council, National Institute of Health Research and UK Aid (grant number MR/T040238/1). All views expressed here are of the authors only.

For peer review only - http://bmjopen.bmj.com/site/about/guidelines.xhtml

Competing interests statement.

The authors declare no competing interests.

References

1 Vos T, Lim SS, Abbafati C, *et al.* Global burden of 369 diseases and injuries in 204 countries and territories, 1990–2019: a systematic analysis for the Global Burden of Disease Study 2019. *Lancet* 2020;**396**:1204–22. doi:10.1016/S0140-6736(20)30925-9

2 Kessler RC, Angermeyer M, Anthony JC, *et al.* Lifetime prevalence and age-of-onset distributions of mental disorders in the World Health Organization's World Mental Health Survey Initiative. *World Psychiatry* 2007;**6**:168–76. Pmid:https://pubmed.ncbi.nlm.nih.gov/18188442/

3 Patton GC, Coffey C, Romaniuk H, *et al.* The prognosis of common mental disorders in adolescents: a 14-year prospective cohort study. *Lancet* 2014;**383**:1404–11. doi:10.1016/S0140-6736(13)62116-9

4 Adolescent Development and Participation, Available: https://www.unicef.org/india/what-we-do/adolescent-development-participation [Accessed 25 June 2021]

5 Gautham MS, Gururaj G, Varghese M, *et al*. The National Mental Health Survey of India (2016): Prevalence, socio-demographic correlates and treatment gap of mental morbidity. *Int J Soc Psychiatry* 2020; **66**(4): 361-372. doi.org/10.1177/0020764020907941

6 Aggarwal S, Berk M. Evolution of adolescent mental health in a rapidly changing socioeconomic environment: a review of mental health studies in adolescents in India over last 10 years. *Asian J Psychiatr* 2015;**13**:3–12. doi:10.1016/j.ajp.2014.11.007

7 Patton GC, Sawyer SM, Santelli JS, *et al.* Our future: a Lancet commission on adolescent health and wellbeing. *Lancet* 2016;**387**:2423–78. doi:10.1016/S0140-6736(16)00579-1

8 Barry MM, Clarke AM, Jenkins R, *et al.* A systematic review of the effectiveness of mental health promotion interventions for young people in low and middle-income countries. *BMC Public Health* 2013;**13**:835. doi:10.1186/1471-2458-13-835

9 Bradshaw M, Gericke H, Coetzee BJ, *et al.* Universal school-based mental health programmes in low- and middle-income countries: A systematic review and narrative synthesis. *Prev Med* 2021;**143**:106317. doi:10.1016/j.ypmed.2020.106317

10 Fazel M, Patel V, Thomas S, *et al.* Mental health interventions in schools in low-income and middle-income countries. *Lancet Psychiatry* 2014;**1**:388–98. doi:10.1016/S2215-0366(14)70357-8

11 Annual State Education Report. ASER 2018 Full report. New Delhi: ASER Centre 2019.

12 Bronfenbrenner U. Ecological systems theory. In: *Six theories of child development: Revised formulations and current issues*. Jessica Kingsley Publishers, 1992:187–249.

13 Deb S, Kumar A, Holden GW, *et al.* School corporal punishment, family tension, and students' internalizing problems: Evidence from India. *School Psychol Int* 2017;**38**:60–77. doi:10.1177/0143034316681378

For peer review only - http://bmjopen.bmj.com/site/about/guidelines.xhtml

14 Jyoti S. Implications of corporal punishment on primary school children. *IOSR J Hum Soc Sci* 2013;**15**:57–61. doi:10.9790/0837-1565761

15 Corporal Punishment in Schools Longitudinal Evidence from Ethiopia, India Peru and Viet Nam, 2015. Available: https://www.unicef-irc.org/publications/pdf/CORPORAL%20PUNISHMENT%20IDP2finalrev.pdf

16 Arun P, Garg R, Chavan BS. Stress and suicidal ideation among adolescents having academic difficulty. *Ind Psychiatry J* 2017;**26**:64–70. doi:10.4103/ipj.ipj_5_17

17 Deb S, Strodl E, Sun J. Academic Stress, Parental Pressure, Anxiety and Mental Health among Indian High School Students. *Int J Psychol Behav Sci* 2015;**5**:26–34. 10.5923/j.ijpbs.20150501.04

18 Venkataraman S, Patil R, Balasundaram S. Stigma toward mental illness among higher secondary school teachers in Puducherry, South India. *J Family Med Prim Care* 2019;**8**:1401–7. doi:10.4103/jfmpc.jfmpc_203_19

19 Parikh N, Parikh M, Vankar G, *et al.* Knowledge and attitudes of secondary and higher secondary school teachers toward mental illness in Ahmedabad. *Indian J Soc Psychiatry*;**32**:56. doi:10.4103/0971-9962.176770

20 Venkataraman S, Patil R, Balasundaram S. Why mental health literacy still matters: a review. *Int J Community Med Public Health* 2019;**6**:2723–9. doi:10.18203/2394-6040.ijcmph20192350

21 Kumar A. Cultures of learning in developing education systems: Government and NGO classrooms in India. *Int J of Educ Res* 2019;**95**:76–89. doi:10.1016/j.ijer.2019.02.009

22 Parikh R, Michelson D, Sapru M, *et al.* Priorities and preferences for school-based mental health services in India: a multi-stakeholder study with adolescents, parents, school staff, and mental health providers. *Glob Ment Health (Camb)* 2019;**6**. doi:10.1017/gmh.2019.16

23 Health promoting schools. https://www.who.int/health-topics/health-promoting-schools#tab=tab_1 [(accessed 25 June 2021]).

24 Srikala B, Kishore KKV. Empowering adolescents with life skills education in schools – School mental health program: Does it work? *Indian J Psychiatry* 2010;**52**:344–9. doi:10.4103/0019-5545.74310

25 Kumar D, Bharath S, Hirisave U, *et al.* School mental health programs in India: Current status and future directions. In: Weist MD, Kutcher S, Wei Y, eds. *School Mental Health: Global Challenges and Opportunities*. Cambridge University Press 2015: 95–104. doi:10.1017/CBO9781107284241.009

26 Shinde S, Weiss HA, Varghese B, *et al.* Promoting school climate and health outcomes with the SEHER multi-component secondary school intervention in Bihar, India: a cluster-randomised controlled trial. *Lancet* 2018;**392**:2465–77. doi:10.1016/S0140-6736(18)31615-5

27 Gujar K, Pingale V. School mental health programme in Maharashtra - Today and way ahead. *Ann Indian Psychiatry*;**1**:4. doi:10.4103/aip.aip_14_17

For peer review only - http://bmjopen.bmj.com/site/about/guidelines.xhtml

28 The State of the World's Children 2011: Adolescence - An age of opportunity. UNICEF DATA, 2011. Available: https://data.unicef.org/resources/the-state-of-the-worlds-children-2011-adolescents-an-age-of-opportunity/ [Accessed 8 May 2021].

29 Roy K, Shinde S, Sarkar BK, *et al.* India's response to adolescent mental health: a policy review and stakeholder analysis. *Soc Psychiatr Epidemiol* 2019;**54**:405–14. doi:10.1007/s00127-018-1647-2

30 Werner-Seidler A, Perry Y, Calear AL, *et al.* School-based depression and anxiety prevention programs for young people: A systematic review and meta-analysis. *Clin Psychol Rev* 2017;**51**:30–47. doi:10.1016/j.cpr.2016.10.005

31 Fazel M, Hoagwood K, Stephan S, *et al.* Mental health interventions in schools 1. *Lancet Psychiatry* 2014;**1**:377–87. doi:10.1016/S2215-0366(14)70312-8

32 Rajaraman D, Travasso S, Chatterjee A, *et al.* The acceptability, feasibility and impact of a lay health counsellor delivered health promoting schools programme in India: a case study evaluation. *BMC Health Serv Res* 2012;**12**:127. doi:10.1186/1472-6963-12-127

33 Shinde S, Weiss HA, Khandeparkar P, *et al.* A multicomponent secondary school health promotion intervention and adolescent health: An extension of the SEHER cluster randomised controlled trial in Bihar, India. *PLoS Med* 2020;**17**:e1003021. doi:10.1371/journal.pmed.1003021

34 Craig P, Dieppe P, Macintyre S, *et al.* Developing and evaluating complex interventions: the new Medical Research Council guidance. *BMJ* 2008;**337**:a1655. doi:10.1136/bmj.a1655

35 Wight D, Wimbush E, Jepson R, *et al.* Six steps in quality intervention development (6SQuID). *J Epidemiol Community Health* 2016;**70**:520–5. doi:10.1136/jech-2015-205952

36 World Health Organization. (2007). *Task shifting to tackle health worker shortages.* Geneva: WHO.

37 Movsisyan, A., Arnold, L., Evans, R. et al. Adapting evidence-informed complex population health interventions for new contexts: a systematic review of guidance. *Implement Sci* 2019; **14**:105. https://doi.org/10.1186/s13012-019-0956-5

38 Perera, C., Salamanca-Sanabria, A., Caballero-Bernal, J. et al. No implementation without cultural adaptation: a process for culturally adapting low-intensity psychological interventions in humanitarian settings. *Confl Health* 2020;**14**: 46. https://doi.org/10.1186/s13031-020-00290-0

39 Hugh-Jones S, Prabhu S, Lakshman KA, *et al.* The effectiveness of interventions to improve secondary school teachers' mental health literacy: a protocol for an umbrella review CRD42021250458. Published Online First: 2021. http://www.crd.york.ac.uk/PROSPERO/display_record.asp?ID=CRD42021250458

40 Hugh-Jones S, Prabhu S, Warriner L, *et al.* The effectiveness of universal school interventions for prevention or treatment of adolescent anxiety and depression: a protocol for an umbrella review. CRD42021248592. Published Online First: 2021. http://www.crd.york.ac.uk/PROSPERO/display_record.asp?ID=CRD42021248592

For peer review only - http://bmjopen.bmj.com/site/about/guidelines.xhtml

41 Hugh-Jones S, Prabhu S, Podiya JK, *et al.* The effectiveness of interventions to improve school climate in secondary schools: a protocol for an umbrella review. CRD42021251790. Published Online First: 2021. http://www.crd.york.ac.uk/PROSPERO/display_record.asp?ID=CRD42021251790

42 Hugh-Jones S, Prabhu S, Nag R, *et al.* The effectiveness of interventions to improve parent mental health literacy: a protocol for an umbrella review. CRD42021252006. Published Online First: 2021. http://www.crd.york.ac.uk/PROSPERO/display_record.asp?ID=CRD42021252006

43 Boustani M, Daleiden E, Bernstein A, *et al.* Using relevance mapping methodology to design an adolescent mental health intervention in India. *Glob Health Action* 2020; **13**:1. doi: 10.1080/16549716.2020.1775062

44 Hudson KG, Lawton R, Hugh-Jones S. (2020). Factors affecting the implementation of a whole school mindfulness program: a qualitative study using the consolidated framework for implementation research. *BMC Health Serv Res* 2020;**20**: 1-13. https://doi.org/10.1186/s12913-020-4942-z

45 Damschroder LJ, Aron DC, Keith RE, *et al.* Fostering implementation of health services research findings into practice: a consolidated framework for advancing implementation science. *Implement Sci* 2009;**4**:50. doi:10.1186/1748-5908-4-50

46 Stirman, S.W., Miller, C.J., Toder, K. et al. Development of a framework and coding system for modifications and adaptations of evidence-based interventions. *Implement Sci* 2013; **8**: 65). https://doi.org/10.1186/1748-5908-8-65

47 Bernal G, Sáez-Santiago E. Culturally centered psychosocial interventions. *J Community Psychol* 2006;**34:**121–32. doi:https://doi.org/10.1002/jcop.20096

48 Stirman, SW, Baumann AA, Miller, CJ. The FRAME: an expanded framework for reporting adaptations and modifications to evidence-based interventions. *Implement Sci2019;* ***14***(1), 1-10.

49 Lancaster GA, Dodd S, Williamson PR. Design and analysis of pilot studies: recommendations for good practice. *Jour Eval Clin Practice* 2004;10:307-312

50 Goodman R. Psychometric Properties of the Strengths and Difficulties Questionnaire. *J Am Acad Child Adolesc Psychiatry* 2001;**40**:1337–45. doi:10.1097/00004583-200111000-00015

51 Chorpita BF, Moffitt CE, Gray J. Psychometric properties of the Revised Child Anxiety and Depression Scale in a clinical sample. *Behav Res Ther* 2005;**43**:309–22. doi:10.1016/j.brat.2004.02.004

52 Wolpert M, Cheng H, Deighton J. Measurement Issues: Review of four patient reported outcome measures: SDQ, RCADS, C/ORS and GBO - their strengths and limitations for clinical use and service evaluation. *Child Adolesc Ment Health* 2015;**20**:63–70. doi:10.1111/camh.12065

53 Hugh-Jones S, Pert K, Kendal S*, et al.* MindMate2U: Development and feasibility study of a co-designed school-based mHealth intervention for symptomatic adolescents. JMIR Preprints. 22/04/2021:29834

For peer review only - http://bmjopen.bmj.com/site/about/guidelines.xhtml

54 Berry V, Axford N, Blower S, *et al.* The Effectiveness and Micro-costing Analysis of a Universal, School-Based, Social–Emotional Learning Programme in the UK: A Cluster-Randomised Controlled Trial. *School Ment Health* 2016;**8**:238–56. doi:10.1007/s12310-015-9160-1

55 Brazier J, Ratcliffe J, Saloman J, *et al. Measuring and Valuing Health Benefits for Economic Evaluation*. Oxford University Press 2017.

56 Stevens K. Developing a descriptive system for a new preference-based measure of health-related quality of life for children. *Qual Life Res* 2009;**18**:1105–13. doi:10.1007/s11136-009-9524-9

57 Ravens-Sieberer U, Wille N, Badia X, *et al.* Feasibility, reliability, and validity of the EQ-5D-Y: results from a multinational study. *Qual Life Res* 2010;**19**:887–97. doi:10.1007/s11136-010-9649-x

58 Herdman M, Gudex C, Lloyd A, *et al.* Development and preliminary testing of the new five-level version of EQ-5D (EQ-5D-5L). *Qual Life Res* 2011;**20**:1727–36. doi:10.1007/s11136-011-9903-x

59 Al-Janabi H, Flynn TN, Coast J. Development of a self-report measure of capability wellbeing for adults: the ICECAP-A. *Qual Life Res* 2012;**21**:167–76. doi:10.1007/s11136-011-9927-2

60 Gale NK, Heath G, Cameron E, *et al*. Using the framework method for the analysis of qualitative data in multi-disciplinary health research. BMC Med Res Methodol 2013; **13**: 117 (2013). https://doi.org/10.1186/1471-2288-13-117

61 Terry G, Hayfield N, Clarke V, *et al.* Thematic analysis. *The Sage Handbook of Qualitative Research in Psychology* 2017; pp 17-37.

62 Coast J. Is economic evaluation in touch with society's health values? *BMJ* 2004;**329**:1233–6. doi:10.1136/bmj.329.7476.1233

63 Mirzoev T, Das M, Ebenso B, *et al.* Contextual influences on the role of evidence in health policy development: what can we learn from six policies in India and Nigeria? *Evid Policy 2017;* **13**(1):59-79.

64 Mirzoev T, Green A, Gerein N, *et al.* 2013. Role of evidence in maternal health policy processes in Vietnam, India and China: findings from the HEPVIC project. *Evid Policy* 2013; **9**(4):493-511.

65 Lavis J, Oxman A, Lewin S, *et al.* SUPPORT Tools for evidence-informed health Policymaking (STP). *Health Res Policy Syst 2009;*7(Suppl 1):I1.

66 Green A, Gadsby E. 2007. A framework for evidence-informed health policy-making. In: *Green A, Bennett S, editors. Sound Choices: Enhancing Capacity for Evidence-Informed Health Policy AHPSR Biennual Review*. WHO, Alliance for Health Policy and Systems Research.

67 Global Consensus Statement, Meaningful Adolescent & Youth Engagement, 2018. Available: https://www.who.int/pmnch/mye-statement.pdf?ua=1 [Accessed 25 June 2021].

For peer review only

For peer review only - http://bmjopen.bmj.com/site/about/guidelines.xhtml

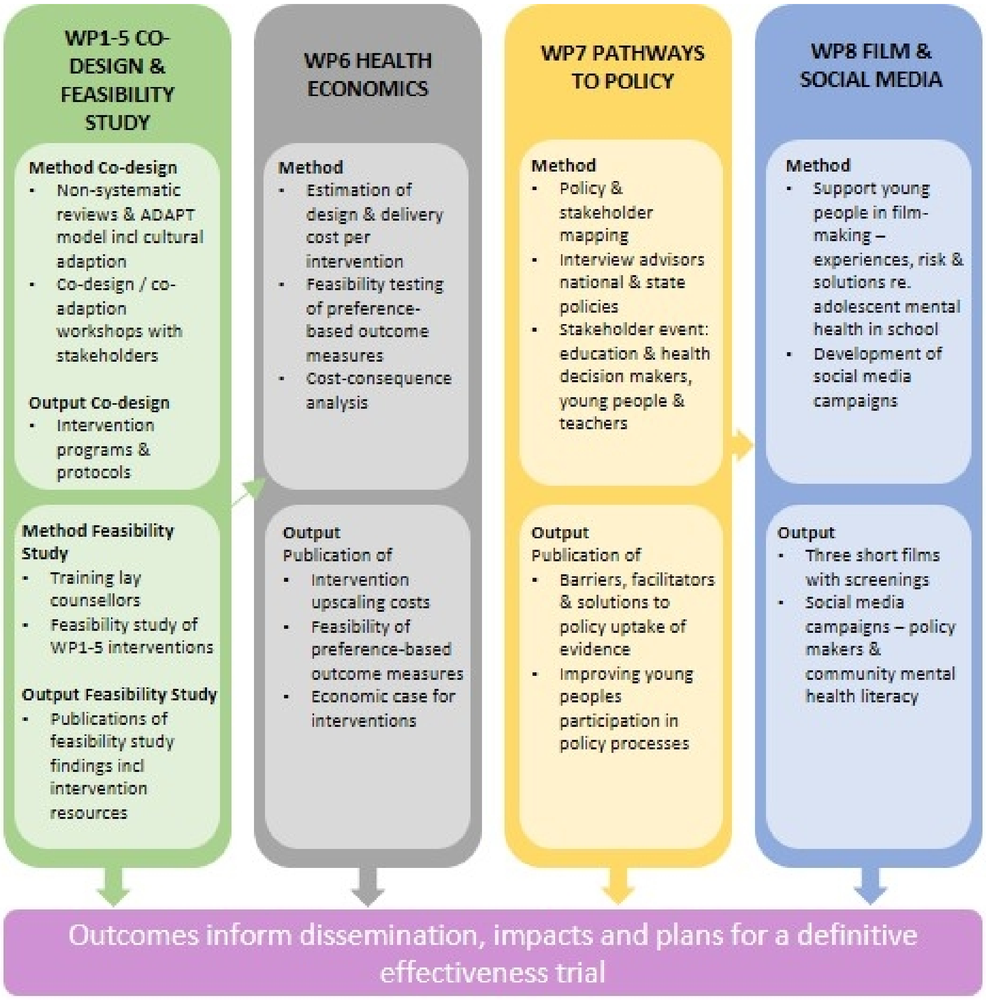

Fig 1 Study work packages

89x90mm (150 x 150 DPI)

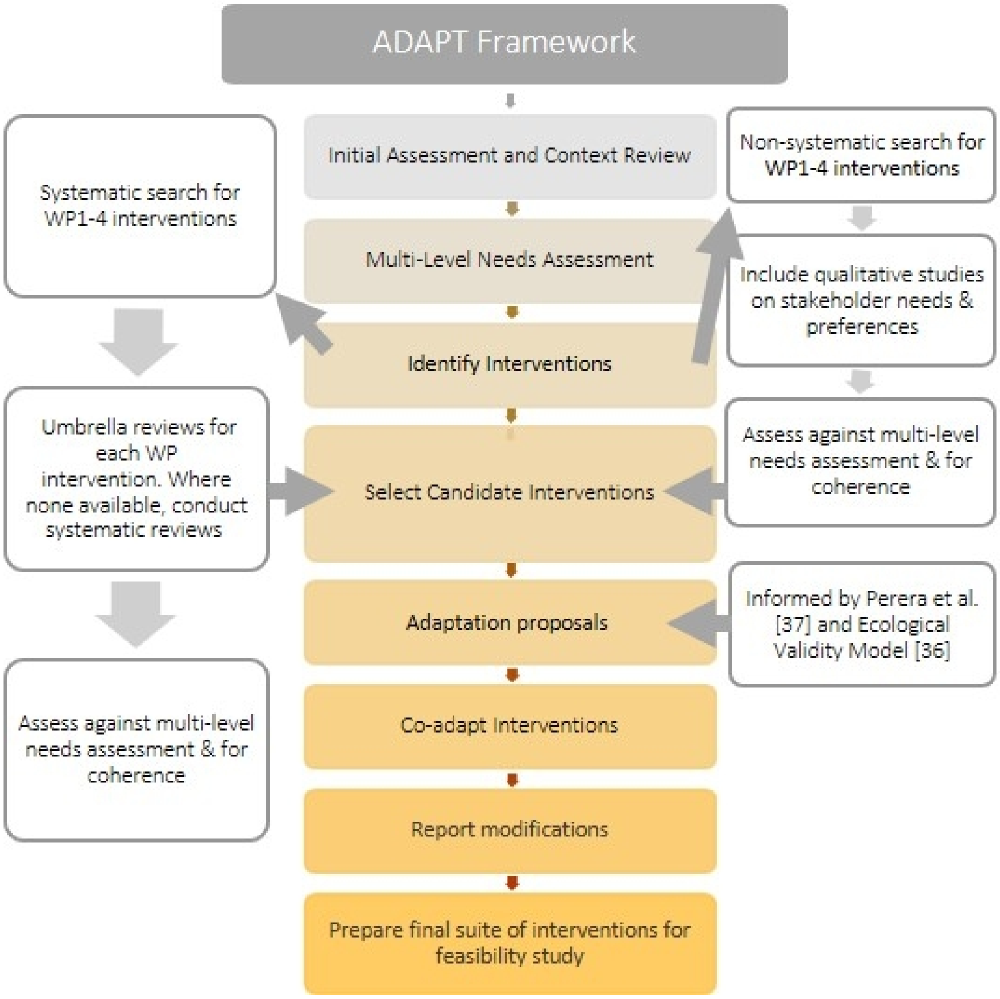

Figure 2 Stages of co-adaptation

90x90mm (150 x 150 DPI)

BMJ Open

Safeguarding Adolescent Mental Health in India (SAMA): study protocol for co-design and feasibility study of a school systems intervention targeting adolescent anxiety and depression in India

Journal:	*BMJ Open*
Manuscript ID	bmjopen-2021-054897.R2
Article Type:	Protocol
Date Submitted by the Author:	22-Feb-2022
Complete List of Authors:	Hugh-Jones, Siobhan ; University of Leeds Faculty of Biological Sciences, Psychology<br>Janardhana, N; NIMHANS Bengaluru, Department of Psychiatric Social Work; National Institute of Mental Health and Neuro Sciences, Department of Psychiatric Social Work<br>Al-Janabi, Hareth; University of Birmingham, Health Economics Unit, School of Health and Population Sciences<br>Bhola, Poornima; National Institute of Mental Health and Neurosciences, Department of Psychiatric Social Work<br>Cooke, Paul ; University of Leeds Faculty of Arts Humanities and Cultures, School of Languages, Cultures and Societies<br>Fazel, Mina; University of Oxford, Warneford Hospital,, Psychiatry; University of Oxford<br>Hudson, Kristian; Bradford Institute for Health Research, Improvement Academy<br>Khandeparkar, Prachi; Sangath<br>Mirzoev, Tolib; London School of Hygiene & Tropical Medicine, Global Health and Development<br>Venkataraman, Surendran; Indira Gandhi Medical College and Research Institute<br>West, Robert; University of Leeds, Leeds Institute of Health Sciences<br>Mallikarjun, Pavan; University of Birmingham
**Primary Subject Heading**:	Mental health
Secondary Subject Heading:	Public health, Global health
Keywords:	MENTAL HEALTH, PUBLIC HEALTH, PREVENTIVE MEDICINE

SCHOLARONE™
Manuscripts

Safeguarding Adolescent Mental Health in India (SAMA): study protocol for co-design and feasibility study of a school systems intervention targeting adolescent anxiety and depression in India.

Hugh-Jones, Siobhan[a*]., Naidu, Janardhan[b]., Al-Janabi, Hareth[c]., Bhola, Poornima [b]., Cook, Paul[a]., Fazel, Mina[d]., Hudson, Kristian[e]., Khandeparkar, Prachi[f]., Mirzoev, Tolib[g]., Venkataraman, Surendran[h]., West, Robert[a]., Mallikarjun, Pavan.[c]

[a] University of Leeds, Leeds, UK

[b] National Institute of Mental Health and Neurosciences, Bangalore, India

[c] University of Birmingham, Birmingham, UK

[d] University of Oxford, Oxford, UK

[e] Bradford Institute of Health Research, Bradford, UK

[f] Sangath, Goa, India

[g] London School of Hygiene and Tropical Medicine, London, UK

[h] Indira Gandhi Medical College and Research Institute, Pondicherry, India

*Corresponding author: Dr Siobhan Hugh-Jones, School of Psychology, University of Leeds, LS2 9JT. E: S.hugh-jones@leeds.ac.uk. Tel: 0113 343 5744 Fax: 0113 343 549

Keywords: adolescent, anxiety, depression, mental health, schools

Word count: 4848

For peer review only

For peer review only - http://bmjopen.bmj.com/site/about/guidelines.xhtml

Abstract

Introduction

Symptoms of anxiety and depression in Indian adolescents are common. Schools can be opportune sites for delivery of mental health interventions. India, however, is without a whole-school mental health approach. This article describes the study design for the Safeguarding Adolescent Mental Health in India (SAMA) project. The aim of SAMA is to co-design and feasibility test a suite of multi-component interventions across the intersecting systems of adolescents, schools, families and their local communities in India.

Methods and analysis

Our project will co-design and feasibility test four interventions to run in parallel in eight schools (three assigned to waitlist) in Bengaluru and Kolar in Karnataka, India. The primary aim is to reduce the prevalence of adolescent anxiety and depression. Co-design of interventions will build on existing evidence and resources. Interventions for adolescents at school will be universal, incorporating curriculum and social components. Interventions for parents and teachers will target mental health literacy, and also for teachers, training in positive behaviour practices. Intervention in the school community will target school climate to improve student mental health literacy and care. Intervention for the wider community will be via adolescent-led films and social media. We will generate intervention cost estimates, test outcome measures and identify pathways to increase policy action on the evidence.

Ethics and dissemination

Ethical approval has been granted by the National Institute of Mental Health Neurosciences Research Ethics Committee (NIMHANS/26th IEC (Behv Sc Div / 2020/2021)) and the University of Leeds School of Psychology Research Ethics Committee (PSYC-221). Certain data will be available on a data sharing site. Findings will be disseminated via peer-reviewed journals and conferences.

For peer review only - http://bmjopen.bmj.com/site/about/guidelines.xhtml

Article Summary

Strengths and Limitations

1. A key strength of this study is its focus on depression and anxiety prevention which are common mental health conditions among Indian adolescents.
2. The study utilises a public mental health systems approach, which adopts a 'person-in-context' perspective and recognises multiple determinants of mental health.
3. Extensive co-design of interventions will promote cultural relevance and acceptability by key stakeholders, including adolescents, teachers, parents and school communities.
4. The Covid-19 pandemic makes this research more pertinent but may affect recruitment of the schools / adolescents or may lead to participation bias if the study processes are conducted online by excluding those with limited access or who lack technical skills.
5. The opt-in approach to consent may lead to participation bias.

For peer review only

Introduction

Mental health conditions including depression and anxiety disorders are among the top ten causes of illness and disability in adolescents.[1] Half of all lifelong mental-health conditions have their onset in adolescence. [2] Poor mental health in adolescence is associated with poor physical health and lifelong disadvantage, especially if education is disrupted, impacting occupational and social trajectories.[3]

India has the largest adolescent population in the world (253 million).[4] An estimated 9.8 million Indian 13–17-year-olds (pooled prevalence of 7.3%) have a clinical mental-health condition [5] but levels seem considerably higher in urban areas and among school-going adolescents. [6] Although there is limited data on the treatment gap in adolescents, the overall treatment gap for mental disorders in India is around 90%. [5] The treatment gap and the effectiveness and cost-effectiveness of early intervention are driving investment into public mental health in efforts to develop scalable programmes that can reach adolescents before their symptoms progress to levels of a clinical disorder.[5] Tackling preventable and treatable health conditions, and improving the quality of education for adolescents, present the single best investments for health and wellbeing a low-and-middle income (LMIC) country can make. [7]

Schools have tremendous potential as platforms for public mental-health interventions, which can secure positive effects on mental health, including a reduction in anxiety and depression, and which can be delivered, sustained and scaled in LMICs. [7] School provision can increase access to interventions over and above existing mental healthcare systems, which in LMICs, are typically fragmented, under-resourced, and not tailored to adolescents. [8–10] Improving school experience may also promote longer participation in education, which reduces a number of risks for girls in particular. [8] The Lancet commission on adolescent wellbeing calls for investment in school mental health, bolstered by the rise in school attendance [8] including in India (97% enrolment). [11] Although some adolescent mental health programs exist in India, there is as yet no integrated, evidence-based, whole school mental health approach for adolescents.

Our project responds to this need by identifying existing school interventions which can be integrated, tailored and culturally adapted into a systems approach [12] in India through a process of co-production with stakeholders. Given the multiple determinants of adolescent mental health in India, [5] interventions will need to target individual and contextual factors. In India, a key contextual determinant of adolescent mental health is the nature of schooling, including the use of corporal punishment [13–15], extreme academic pressure [16,17], a lack of mental health literacy among adolescents, teachers and parents, [18–20] prevailing mental

For peer review only - http://bmjopen.bmj.com/site/about/guidelines.xhtml

health stigma and bullying,[21] poorly supported staff, often with large class sizes and a lack of mental health support within schools.[22] Although the Government of India endorsed the WHO Health Promoting School model (2007) [23] progress on school mental health has been slow. To date, school initiatives in India have mostly targeted physical health and life skills.[24–27] There is an urgent need to accelerate improvements in mental health in Indian schools, from a safety and rights perspective [28], to reduce school risks to adolescent mental health and to improve population health. India has limited evidence-based, scalable school programmes, endorsed by policy, to support adolescent mental health. This is a critical care gap. [5,29] Our study aims to contribute to the evidence base about what works in Indian schools to support adolescent mental health.

There is some evidence for school-based universal mental interventions for depression and anxiety prevention, and mental health promotion, in High-Income Countries (HICs) [30,31]. The evidence in LMICs is promising though limited [8–10]. Three whole-school health and life-skills programmes with small mental health components have been tested in India.[24,26,32,33] One did not report any beneficial clinical outcomes [24] and one has yet to establish effectiveness. [32] indicating that life-skills interventions may be insufficient to impact adolescent anxiety and depression. The third is a health promotion programme (SEHER) focusing on school climate, and targeting physical and sexual health, bullying, gender equality and depressive symptoms. It focused on promoting adolescents' social and problem-solving skills, engaging adolescents, teachers, and parents in school-level decision-making and delivering factual knowledge about health and risk behaviours to the school community. [26] Outcomes were positive, including for depressive symptoms, with benefits extended up to two years [33]. It did not, however, target anxiety nor include broad mental health promotion. This is a critical gap given the prevalence of anxiety disorders in adolescence and its co-morbidity with depression.

Our study **S**afeguarding **A**dolescent **M**ental he**A**lth in India (SAMA) builds on learning from SEHER in terms of the value and acceptability of whole school, multi-component interventions in India schools and the contribution of school climate to adolescent well-being. Project SAMA extends SEHER in several ways. Our health objective is to reduce symptoms of both anxiety and depression in symptomatic adolescents and to promote the wellbeing all school-going adolescents. We will co-design, and feasibility test, a suite of school interventions in India targeting multiple risks and protective factors across the school system. We extend SEHER by including co-designed interventions targeting teachers and parents, as well as an adolescent mental health psychoeducation intervention. We also augment the school climate intervention of SEHER via co-production. The SAMA study will take place between January 2021 and December 2023.

For peer review only - http://bmjopen.bmj.com/site/about/guidelines.xhtml

Method and analysis

Study design

SAMA will run over 36 months and will deliver 8 work packages (WP) (see Figure 1).

WP1-4 are intervention co-design / co-adaptation and feasibility studies following MRC guidance [34] and the Six Steps in Quality Intervention Development 6SQuID [35], targeting adolescents (14–15 years), teachers, the school community and parents (Table 1). WP5 is implementation research on these interventions and WP6 is an exploratory cost-effectiveness study of the interventions. WP7 and 8 target policy and local community systems respectively. Year 1 will focus on co-design and co-adaptation of interventions via WP1-5. Years 2 and 3 will include a feasibility study of the adapted interventions alongside WP6 and 7. WP8 will run across all three years.

SAMA will work with adolescents as partners in all stages of the project. In addition to the adolescents involved as participants in co-design, we will have a Youth Advisory Board who will be consulted on all project components, a subset of whom will form adolescent film crews and drive the social media campaigning (both WP8). We will have three other advisory boards (Steering Group, Scientific Advisory Board, Ethics Oversight Committee).

----Figure 1: Study work packages----

For WP1-4, some broad interventions requirements have been defined in advance of co-design, including the aims and primary and secondary outcomes. These are shown in Table 1. It is possible that co-design discussions may lead to the inclusion of additional outcome measures. All interventions will be delivered by external lay counsellors. Use of lay counsellors can represent effective task-shifting task, an approach endorsed by the World Health Organization in low-resource settings. [36] Task-shifting involves assigning tasks and responsibilities from more specialised to less specialised groups and requires clearly defined roles with effective training and supervision. There is some evidence that this delivery approach is superior and preferred to teacher-delivered interventions, and peer educator programmes, at least for adolescent mental health programmes. [22] [26] [32] Notably, the cluster randomised trial of SEHER (a multicomponent school health promotion program) conducted across 75 schools in Goa was found to be effective when delivered by lay counsellors but not teachers. [26] There are additional benefits of having a small team of lay counsellors working closely in partnership with schools. These include each lay counsellors having a co-counsellor to work alongside to deliver SAMA, counsellors being able to get to

For peer review only - http://bmjopen.bmj.com/site/about/guidelines.xhtml

know particular schools well (i.e. their systems, culture), having constancy in the lay counsellors so school staff and students can build familiarity and trust, and having a defined conduit for communication about SAMA within schools and between school and the study team.

Table 1: Intervention aims and requirements defined in advance

WP	Intervention	Requirements defined prior to co-design	Primary outcome	Secondary outcomes (include but not restricted to)
1	Whole school curriculum adolescent mental health programme	15-16y (Grade 9) as advised by schools<br><br>6-8 weeks duration	Symptoms of anxiety and depression	Mental health literacy, stress, self-esteem, resilience, quality of life, school attendance and performance.
2	Teacher education programme	Low intensity<br><br>Suitable for all levels of staff	Teacher mental health literacy	Teacher attitude towards and use of positive behaviour management practices.
3	School climate intervention	Up to 6 months	School climate	Mental health literacy, perceived stigma, perceived support
4	Parent mental health literacy intervention	Low intensity	Parent mental health literacy	Requests for more mental health information / support / referrals

For peer review only

School recruitment

We will recruit eight schools in Kolar and Bangalore to participate in both the co-design and feasibility stages of SAMA (WP1–6). School recruitment will be facilitated by the Karnataka state primary and secondary education ministry and the Karnataka state secondary school Headmaster's Association. We will recruit two school types (government and low-cost private) to identify school factors that may affect feasibility. Schools will be eligible to take part in SAMA if they endorse the delivery of WP1–6 in their schools, including participation in co-design. WP1 intervention feasibility testing will be with a new Grade 9 cohort not involved in co-design.

Year 1 Co-design / co-adaptation of interventions

Co-design will proceed in stages (see Figure 2) and will include co-adaptation of existing interventions as well as design of new components where needed (e.g. content, implementation, safeguarding). We begin with co-adapting interventions before identifying the need for new co-designed elements. Our approach to co-adaptation is informed by

For peer review only - http://bmjopen.bmj.com/site/about/guidelines.xhtml

phases 1–3 of the ADAPT model for introducing complex public health interventions into new settings [37] and also by Perera et al.'s approach [38] to adapting low-intensity psychological interventions into new cultural settings. WP5 runs alongside WP1–4 and aims to identify implementation facilitators and barriers for all intervention components. As well as adapting interventions, we will co-produce with adolescent and stakeholders a whole-school safeguarding protocol tailored to the interventions.

----Figure 2: Stages of co-adaptation / co-design---

Phase 1: Exploration of needs and existing interventions

Initial assessment

A desk review will be conducted to gather knowledge on the local context and research evidence relevant to our project aims, including identification of any existing best practice and policy requirements. This will feed into an evidence-based, multi-level needs assessment of end-users, families, schools and regional / state systems to sensitise our selection of interventions and implementation approach.

Intervention selection & exploration

We aim to identify interventions that are likely to be acceptable, feasible and efficacious in our target context to meet our project aims. We will search for interventions with the potential (after co-adaptation) to impact at least the primary outcome for each WP (Table 1). As per Figure 2, a non-systematic search will be conducted first to collate or identify interventions which are known to the team in India, referenced in Indian education or mental health policies or referenced by Indian or global organisations (e.g. Save the Children, WHO). This will be followed by a systematic search. Umbrella review protocols for these are registered on Prospero [39–42] and reviews are nearing completion. Considering search returns from both the non-systematic and systematic searched, WP1–4 leads will propose candidate interventions to be taken to Phase 2. Whilst established effectiveness of an intervention is important, we also need to consider the coherence, integration, implementation and scalability of interventions for the local context. [43] Proposals will be reviewed by the research team and project advisory boards, which include in-country adolescents, schools and mental health professionals as well as the team which led SEHER [26]. Amendments to proposals will be made where necessary.

For peer review only - http://bmjopen.bmj.com/site/about/guidelines.xhtml

This phase includes work on implementation protocols (WP5). We will build on a UK framework for a whole-school mental health intervention [44] based on the Consolidated Framework for Implementation Research (CFIR) [45]. The framework will be iteratively developed as SAMA interventions are finalised, drawing on existing implementation evidence from India, including the SEHER study [26], and through our co-adaptation processes.

Phase 2: Preparation for co-design

Identification of mismatches and model development

Candidate interventions will be assessed against our multi-level needs assessment; those failing to meet significant need will be rejected. We will then develop a model of how the final interventions could work across systems before identifying the aspects (across surface and deep levels [46]) which are most critical to take forward to co-design / co-adaptation. Decisions here will be informed by the approach of Perera et al. [38] and their use of the Ecological Validity Model [47]. This will lead to the generation of adaptation hypotheses, that is, our proposals for which intervention aspects will need to be culturally adapted. The Ecological Validity Model prompts directs consideration of the language, persons, metaphors, content, concepts, goals, methods and context (which includes implementation) of the intervention to be adapted. We will supplement these proposals with co-design needs, that is, new features which need to be created because of the systems approach or local school context. Co-adaptation and co-design proposals will be finalised in consultation with our youth and advisory boards.

Co-adaptation and co-design

Our aim in this stage is to optimise the acceptability, feasibility, efficacy and meaningful evaluation of the suite of interventions by involving end-users and stakeholders in intervention adaptation and its associated implementation approach. We will host co-design / co-adaptation workshops for each WP intervention, each involving adolescents (14–16y, single gendered groups with more females), parents, schools (including teachers and institute heads) and key stakeholders in separate workshops. WP2 co-design will additionally involve national organisations (e.g. State Education & Health Department, Teachers of India, ChildLine India, and Save the Children). WP4 co-design will begin with a stakeholder event to identify community-led solutions to reaching parents. Creative evaluation methods will be devised during co-design to be sensitive to low literacy levels, low chance of measure completion and desirability bias. Implementation barriers, facilitators and developing implementation protocol will be a standing item for consultation in co-design workshops. Co-

For peer review only - http://bmjopen.bmj.com/site/about/guidelines.xhtml

design / co-adaptation workshops will include a range of age- and role-appropriate discussions and activities centering on the co-adaptation proposals developed in the previous stage.

Phase 3: Undertaking Modifications

We will modify the interventions based on co-adaptation and co-design workshop outputs. We will utilise the expanded FRAME approach to report modifications to interventions. [48] This approach documents when, why and how modifications were made, and differentiates cultural adaptation from adaptations made for other reasons. Proposed modifications will be reviewed by the full team to ensure modified interventions still meet the multi-level needs assessment, retain active ingredients and are coherent as a suite of interventions. Modifications and final prototype interventions will be reviewed by our youth and advisory boards as well as a board of in-country mental health professionals and education sector representatives to ensure they meet state and national requirements. Once finalised, we will produce for each WP intervention a logic model, programme manual, intervention resources, lay counsellor training and supervision protocol, implementation and evaluation protocols.

Year 2 Feasibility testing

Lay counsellors will be from the local areas, have at least a Masters qualification in psychology or a related discipline and experience in working with young people and / or schools. Prior to the launch of the feasibility study, they will receive full training over three months by a clinical psychologist on the team, supported by another team member experienced in training lay counsellors. Trainees will deliver multiple mock sessions of intervention delivery to support readiness for delivery in schools and quality control. The feasibility study will run in eight schools, with three randomly assigned to waitlist. The four interventions will run in parallel in schools. Lay counsellors will complete weekly monitoring logs of intervention delivery in each school, including logging of modifications made (as per FRAME [48]) and will have weekly supervision by clinical team members. Fidelity monitoring will be undertaken by a team of trained data collection assistants who have been heavily involved in the intervention development stage and will be highly familiar with the implementation protocol. Fidelity monitoring reports will be discussed frequently with the research manager to identify needs for lay counsellor action / support or intervention modification (see WP5).

For peer review only - http://bmjopen.bmj.com/site/about/guidelines.xhtml

We have established preliminary feasibility indicators for each intervention, which will be updated when interventions are finalised. Feasibility indicators which will be common across WP1–4 interventions are: meeting recruitment and retention rates, delivery as intended, measure completion and no significant adverse effects. We will adopt a 'traffic-light' system for recruitment and retention as follows: Red – if rates below 50% (say) then will not proceed; Amber – if rates 50-65% then proceed if there are plans to improve; Green – above 65% proceed to definitive trial. Mixed-methods process and evaluation data will be secured for all interventions from adolescents, teachers, parents and lay counsellors to explore: perceived usefulness of the interventions; implementation challenges; unintended negative effects; safeguarding challenges; the process of change and intervention development needs. Evaluation outcomes will inform intervention improvements and parameters for a definitive trial.

Sample size calculation

The planned sample for the feasibility study by intervention is detailed in Table 2. The minimum recommended sample size for a feasibility study is 30. [49] The smallest group of participants are teachers (for the WP2 intervention). To secure a sample of 30, we will need to recruit a minimum of 8 schools, from which we will then be able to recruit approximately n=960 Grade 9 adolescents (for WP1 intervention). Given this adolescent sample size, we have been able to calculate power in relation to WP1, where a key aim is to test two candidate primary outcomes measures - the Strengths and Difficulties Questionnaire (SDQ) [50] and the Revised Child Anxiety and Depression Scale (RACDS) [51]. SDQ is an emotional and behavioural screening tool, culturally validated in India and used widely in adolescent mental health research. RCADS measures anxiety and depression and demonstrates excellent sensitivity-to-change. A translated RCADS has been used with Indian populations [52] but full cultural validity needs to be established. As this will be an important tool to strengthen Indian mental health research, we will conduct a full validation study in parallel to the co-design stage. We have determined via a UK school feasibility trial [53] that the change in SDQ had a standard deviation of 2.8 points. Assuming 80% recruitment, comparing the mean changes in SDQ from 480 participants from the intervention group with 288 from the waitlist group using a 1% significance level has more than 98% power to detect a difference of 1 point on the SDQ scale. Around 960 adolescents will be approached for WP1. At 65% recruitment, the power remains over 95%. This calculation is based on an analysis of variance approach, that is, regressing the change in SDQ upon the intervention status (intervention or waiting list). The power achieved from an

For peer review only - http://bmjopen.bmj.com/site/about/guidelines.xhtml

analysis of covariance approach, which regresses the final SDQ score upon baseline and intervention status, should be similar. Should the mechanism of the WP1 intervention be similar across population subgroups, we can therefore show good power. There may, however, be differences in the operation of the intervention between subgroups, such as government versus private schools. In that case, assuming an even division of samples, the power becomes 85% with 80% recruitment and 78% with 65% recruitment. A significance level of 1% is used rather than 5% since the sensitivity of the outcome measures is sought to be compared – that is, the SDQ will be compared in sensitivity in this setting with the R-CADS.

Table 2: Planned sample size for each intervention in the feasibility study

WP	Intervention	N	Sample
1	Universal curriculum adolescent mental health programme	960	Grade 9 15–16-year-olds from 8 schools (120 per school)
2	Teacher education programme	32	Grade 9 teachers (4 per school)
3	School climate intervention	8 schools	To be defined following intervention selection
4	Parental mental literacy intervention	960 sets of parents	Aim to reach all parents / carers of WP1 participants

WP5 Implementation research

We will test the feasibility of our co-designed implementation approach, which will draw on improvement science. Lay counsellors will be trained and supported to optimise the intervention in situ by logging and overcoming implementation challenges as they arise, whilst keeping fidelity to intervention function. Process evaluations based on interviews / focus groups and implementation assessment tools will be conducted post-intervention with key school staff and other stakeholder as needed depending on what challenges arise. Solutions to implementation challenges will be sought.

WP6 Health economic evaluation

Using a micro (bottom-up) costing strategy [54], separate design and delivery costs per intervention component will be estimated using available data sources (e.g. government ministry data, national pay scales, national earnings surveys) to determine unit costs for personnel time. Any additional primary data needs will be identified. Costs will be presented at the class and school level to promote generalisability. Preference-based outcome health

For peer review only - http://bmjopen.bmj.com/site/about/guidelines.xhtml

and wellbeing measures are needed for economic evaluation [55] but there is limited evidence on their use with adolescents and no evidence of their use feasibility in this context (with adolescents in school-based settings and in India). We will feasibility test candidate measures including CHU-9D [56] and EQ-5D-Y [57] via pre-post administration to 50 intervention adolescents and via 10 think-aloud interviews to identify completion challenges (a satisfactory sample size given the richness of interview data). We will also administer the EQ-5D-5L [58] and ICECAP-A [59] pre- and post-intervention to 50 parents to determine measure completion and any need for instrument adaptation. These sample sizes are not selected to estimate the significance of a specific outcome, but to balance the desire to not over-burden individuals while still informing completion rates of instruments and items. We will use the cost and outcome data to generate exploratory cost-consequence analyses of the intervention strategies.

WP7 Understanding barriers and opportunities for research to policy uptake

We aim to (i) understand facilitators, barriers and solutions to policy uptake of the evidence on school mental health (in Karnataka); (ii) identify ways of increasing adolescents' participation in policy processes, and (ii) identify the potential for application of solutions to other states. A policy and stakeholder mapping exercise will be undertaken, and then key advisors (n~10) for each of the three policies (two National and one state) [29] will be purposefully identified and interviewed. Insights will be shared at a stakeholder event comprising about 20 education and health decision-makers, adolescents and teachers to generate solutions for policy improvement. Insights will direct a social media campaign for policy action. Evaluation of effectiveness will be based on the number and nature of stakeholders reached, new knowledge on evidence-to-policy barriers and solutions and adolescents' participation.

WP8 Community film making and social media campaign.

Around 15 adolescents (single-gendered groups and more females) will be supported to create 3 short films on experiences and risks around adolescent mental health, the potential for the school to affect mental health, and solutions to safeguard their well-being. Films will be used in WP1-4 for engagement, education, advocacy and impact, and to inform (i) a community and stakeholder's event. Films will be used in WP1-4 for engagement, education, advocacy and impact, via (i) a community and stakeholder's event to screen the films to promote shared understanding and solutions; (ii) a social media education campaign targeting adolescents' mental health literacy; and (iii) an advocacy campaign targeting

For peer review only - http://bmjopen.bmj.com/site/about/guidelines.xhtml

For peer review only

national-level policymakers. Outcomes will include open access films and new knowledge on the feasibility and reach of community filmmaking for health education and advocacy campaigning for school mental health in India.

Data analysis plan

WP1-5 Co-design / co-adaptation

Workshop data will include audio / video recordings, facilitator notes and design materials (e.g. maps, user journeys, reference ranking, annotated implementation and safeguarding protocols). Key workshop moments which informed an adaptation or new design component will be transcribed, supplemented with facilitator notes and accuracy checked by two facilitators. Workshop data from different stakeholder groups per focus (e.g. intervention, implementation, safeguarding) will be integrated by WP leads. They will synthesise proposed modifications and report them as per FRAME [48] to take forward to final approval stages in preparation for the feasibility study (described above).

WP1–5 Feasibility Study

School-level effects will be handled as fixed rather than random effects. Primary analysis will be analysis of covariance where outcomes at three months are regressed upon the baseline value and any relevant covariates/factors as age, sex, school, participant characteristics. The analysis will be supported by a two-level mixed-effects model where measures at two-time points are nested within participants. If further to this analysis, a model is required to examine the relationship between the change in measure over time and the initial measure level, then Bloomqvist's method will be employed. Process evaluations will be analysed using Framework Analysis [60] or Thematic Analysis [61]. The implementation achievements in each school will be quantified per intervention and as a systems approach, and actions associated with success will be identified, drawing on the CFIR [45] and as per Hudson et al. [44].

WP6 Health economic evaluation

For the quantitative validity testing of the outcomes measures, we will calculate completion rates of the measures and items. For the qualitative validity testing. we will code the think-aloud interview transcripts to identify errors or difficulties in responding to items. Mean pre- and post-quality of life scores will be used to estimate quality of life benefits from the

For peer review only - http://bmjopen.bmj.com/site/about/guidelines.xhtml

interventions. These data will be combined with estimates of the intervention costs in a simple cost–consequence analysis [62].

WP7 Understanding barriers and opportunities for research to policy uptake

Data from the stakeholder analysis and document reviews will be continuously triangulated with analysis of transcripts from the in-depth interviews. All qualitative data will be analysed using Framework Approach [58] and guided by the three questions for WP7 and relevant theoretical frameworks for evidence-informed policymaking. [63-66]

For peer review only

Patient and public involvement

Throughout SAMA, listening to adolescents will be central and incorporated in each WP. There are many barriers to adolescent involvement in low resource settings.[31] These can include high levels of mental health stigma as well as cultural differences where adolescent's opinions might be less commonly sought and heard. Our approach to adolescent participation will be informed by the WHO Global Consensus Statement on Meaningful Adolescent & Youth Engagement. [67] Adolescents will be trained and supported to contribute to the project. As well as being involved as participants in the design and evaluation of interventions in WP1-5, other adolescents will sit our Youth Advisory Board to act as 'critical friends' of the project. Adolescent contribution will also be via participatory filmmaking. This is a key platform within and beyond our project for youth voice in India on adolescent mental health and the role of schools in wellbeing. Other stakeholders including parents, teachers, the school community and policymakers will be involved in the co-design and evaluation stages of the study.

Ethics and dissemination

This research is being conducted in community settings and has received ethical approval from the National Institute of Mental Health Neurosciences Research Ethics Committee (NIMHANS/26th IEC (Behv Sc Div / 2020/2021)), and the University of Leeds School of Psychology Research Ethics Committee (PSYC-221). Additional approvals have been granted by the Karnataka state primary and secondary education ministry and the Karnataka Department of State Education Research and Training.

We will seek consent from parents, teachers, school staff and other stakeholders and assent from adolescents. Grade 9 pupils will receive WP1 and 3 but can opt out of data collection. This is to ensure that participants' rights are not undermined by universal delivery. Teachers

For peer review only - http://bmjopen.bmj.com/site/about/guidelines.xhtml

can access WP2 training without consenting to data collection. This is in recognition of the sensitivity of teachers reporting their mental health knowledge or use of harsh discipline practices, and responses will inform our understanding of the acceptability of our data collection measures. Audio/video recording in workshops and for WP8 will require explicit consent. Research data will be kept secure and confidential, with agreed UK–India data management plans and data sharing agreements.

Alongside WP1–5, we will be drafting, and refining through co-design, a stepped response and referral protocol to be assessed as part of the feasibility study. This protocol will draw on national and international guidance, as well as established practice in NIMHANS. Our priorities are response protocols for identification of (i) moderate to serious mental health need or risk (especially suicide risk) and (ii) abuse (especially sexual abuse). The iterative production and field testing of this school mental health safeguarding policy will be an important contribution to the research field as well as to school systems.

Our plans for dissemination include: (i) events – launch, film screening, impact and capacity building events; (ii) website – project website with multiple functionalities to engage, share resources, disseminate findings and in ways that can be sustained beyond the project; (iii) social media (twitter, Instagram ~~and TikTok~~) to reach multiple sectors and adolescents; (iv) project briefs/updates/newsletters (flyers), especially for schools and community organisations; (v) executive reports to key stakeholders (e.g. education and health departments, teacher training colleges, mental health service providers); (vi) sharing the project on global repositories, such as the Mental Health Innovation Network; (vii) using existing communication platforms from University of Leeds Global Challenges project relevant to adolescent wellbeing in LMICs; and (viii) conferences and peer-reviewed publications.

Strengths and Limitations

A key strength of the study is our systems approach [12]. We target multiple risk and protective factors in the school setting as mental health is determined by both individual factors and the circumstances in which adolescents are living their day-to-day lives. A further positive about the study is its focus on anxiety and depression, which are the most common adolescent mental health conditions in India. [5,6] The likely feasibility and efficacy of the SAMA interventions is strengthened by several aspects of study design. Youth voice is central with the use of Youth Advisory Board and filmmaking. We combine learning from SEHER [26,33] with umbrella and systematic reviews of existing school interventions. Extensive investment is planned in intervention co-adaptation / co-design with adolescents,

For peer review only - http://bmjopen.bmj.com/site/about/guidelines.xhtml

schools, parents and key stakeholders, guided by established frameworks, to promote cultural relevance, ownership and acceptability. [37,38] Our whole school approach fosters school ownership, is inclusive, minimises stigma, and acceptability of lay counsellors as delivery agents is established. [33] Inclusion of different school types and locations add to the potential generalisability of findings, and the use of validated and globally used instruments supports the generation of reliable and comparable data, including health economics data. Indian youth involvement, either as co-researchers, co-designers or in advisory panels, remains relatively novel. We will gain new insights into what works in this domain SAMA by supporting youth voice through advocacy via social media, filmmaking and pathways to policy impact. There are a number of study limitations. Study recruitment and engagement may be affected by school disruption from the Covid-19 pandemic, and the opt-in approach to consent may lead to participation bias. The universal approach with Grade 9 students limits the extent to which interventions can be tailored to, for example, other mental health conditions or gender. We will be unable to identify adolescents with neurodevelopmental conditions or particular learning needs, meaning we will not know if they in particular can benefit from the interventions. Our school sampling strategy does not include representation of all possible Indian school structures and cultures.

Discussion

In India, a key contextual determinant of adolescent mental health is the nature of schooling. [13-22] This co-design and feasibility study is an important step towards an integrated, multi-component whole school approach to adolescent mental health in Indian secondary schools. School-delivered well-being programs have the potential to exceed the reach and scalability of mental healthcare systems in LMICs. [8–10] By targeting adolescent anxiety and depression, the interventions produced in SAMA will contribute to public mental health approaches to common mental health conditions in India, and, by promoting well-being factors, can inform health promotion strategies. Unique outcomes of the study include rich data on what Indian schools, and their communities, need and want from a whole school approach to mental health as well insights into what aspects of international programs need particular cultural adaptation. Mental health literacy among Indian adolescents, parents and teachers has been reported as low [18–20]. Our study will generate new knowledge on whether programs developed in other countries, with likely different conceptualisations of mental health and responses to it, can be successfully co-adapted to be acceptable and helpful in Indian schools. By adopting a randomised controlled waitlist design, the study will generate a solid evidence base for progression to a RCT and inform understanding of the acceptability and rigour of data collection instruments and process evaluations in complex

For peer review only - http://bmjopen.bmj.com/site/about/guidelines.xhtml

intervention studies in Indian schools. To date, school initiatives in India have mostly targeted physical health and life skills. [24–27] Drawing upon the expertise of an international, interdisciplinary team, this study aims to contribute to the evidence base about what works in Indian schools to support adolescent mental health, and in particular, to reduce the prevalence and severity of adolescent anxiety and depression.

Contributorship statement: SHJ is guarantor. SHJ, JN, PM, PB, MF, HA, PC, RW and TM designed the study. PM and SHJ prepared the first draft of the manuscript with contribution from HA on health economics, from TM on policy pathways and PC on filmmaking. RW assisted with writing the feasibility study design and data analysis; KH assisted with writing about implementation science; MF, PB and PK assisted with writing about PPIE, lay counsellors, ethics and safeguarding; and SV assisted with writing about mental health literacy. All authors reviewed and approved the final draft.

Funding statement

This work is supported by the UK's Medical Research Council, Economic and Social Research Council, National Institute of Health Research and UK Aid (grant number MR/T040238/1). All views expressed here are of the authors only.

Competing interests statement.

The authors declare no competing interests.

References

1 Vos T, Lim SS, Abbafati C, *et al.* Global burden of 369 diseases and injuries in 204 countries and territories, 1990–2019: a systematic analysis for the Global Burden of Disease Study 2019. *Lancet* 2020;**396**:1204–22. doi:10.1016/S0140-6736(20)30925-9

2 Kessler RC, Angermeyer M, Anthony JC, *et al.* Lifetime prevalence and age-of-onset distributions of mental disorders in the World Health Organization's World Mental Health Survey Initiative. *World Psychiatry* 2007;**6**:168–76. Pmid:https://pubmed.ncbi.nlm.nih.gov/18188442/

3 Patton GC, Coffey C, Romaniuk H, *et al.* The prognosis of common mental disorders in adolescents: a 14-year prospective cohort study. *Lancet* 2014;**383**:1404–11. doi:10.1016/S0140-6736(13)62116-9

4 Adolescent Development and Participation, Available: https://www.unicef.org/india/what-we-do/adolescent-development-participation [Accessed 25 June 2021]

5 Gautham MS, Gururaj G, Varghese M, *et al*. The National Mental Health Survey of India (2016): Prevalence, socio-demographic correlates and treatment gap of mental morbidity. *Int J Soc Psychiatry* 2020; **66**(4): 361-372. doi.org/10.1177/0020764020907941

For peer review only - http://bmjopen.bmj.com/site/about/guidelines.xhtml

6 Aggarwal S, Berk M. Evolution of adolescent mental health in a rapidly changing socioeconomic environment: a review of mental health studies in adolescents in India over last 10 years. *Asian J Psychiatr* 2015;**13**:3–12. doi:10.1016/j.ajp.2014.11.007

7 Patton GC, Sawyer SM, Santelli JS, *et al.* Our future: a Lancet commission on adolescent health and wellbeing. *Lancet* 2016;**387**:2423–78. doi:10.1016/S0140-6736(16)00579-1

8 Barry MM, Clarke AM, Jenkins R, *et al.* A systematic review of the effectiveness of mental health promotion interventions for young people in low and middle-income countries. *BMC Public Health* 2013;**13**:835. doi:10.1186/1471-2458-13-835

9 Bradshaw M, Gericke H, Coetzee BJ, *et al.* Universal school-based mental health programmes in low- and middle-income countries: A systematic review and narrative synthesis. *Prev Med* 2021;**143**:106317. doi:10.1016/j.ypmed.2020.106317

10 Fazel M, Patel V, Thomas S, *et al.* Mental health interventions in schools in low-income and middle-income countries. *Lancet Psychiatry* 2014;**1**:388–98. doi:10.1016/S2215-0366(14)70357-8

11 Annual State Education Report. ASER 2018 Full report. New Delhi: ASER Centre 2019.

12 Bronfenbrenner U. Ecological systems theory. In: *Six theories of child development: Revised formulations and current issues*. Jessica Kingsley Publishers, 1992:187–249.

13 Deb S, Kumar A, Holden GW, *et al.* School corporal punishment, family tension, and students' internalizing problems: Evidence from India. *School Psychol Int* 2017;**38**:60–77. doi:10.1177/0143034316681378

14 Jyoti S. Implications of corporal punishment on primary school children. *IOSR J Hum Soc Sci* 2013;**15**:57–61. doi:10.9790/0837-1565761

15 Corporal Punishment in Schools Longitudinal Evidence from Ethiopia, India Peru and Viet Nam, 2015. Available: https://www.unicef-irc.org/publications/pdf/CORPORAL%20PUNISHMENT%20IDP2finalrev.pdf

16 Arun P, Garg R, Chavan BS. Stress and suicidal ideation among adolescents having academic difficulty. *Ind Psychiatry J* 2017;**26**:64–70. doi:10.4103/ipj.ipj_5_17

17 Deb S, Strodl E, Sun J. Academic Stress, Parental Pressure, Anxiety and Mental Health among Indian High School Students. *Int J Psychol Behav Sci* 2015;**5**:26–34. 10.5923/j.ijpbs.20150501.04

18 Venkataraman S, Patil R, Balasundaram S. Stigma toward mental illness among higher secondary school teachers in Puducherry, South India. *J Family Med Prim Care* 2019;**8**:1401–7. doi:10.4103/jfmpc.jfmpc_203_19

19 Parikh N, Parikh M, Vankar G, *et al.* Knowledge and attitudes of secondary and higher secondary school teachers toward mental illness in Ahmedabad. *Indian J Soc Psychiatry*;**32**:56. doi:10.4103/0971-9962.176770

20 Venkataraman S, Patil R, Balasundaram S. Why mental health literacy still matters: a review. *Int J Community Med Public Health* 2019;**6**:2723–9. doi:10.18203/2394-6040.ijcmph20192350

21 Kumar A. Cultures of learning in developing education systems: Government and NGO classrooms in India. *Int J of Educ Res* 2019;**95**:76–89. doi:10.1016/j.ijer.2019.02.009

For peer review only - http://bmjopen.bmj.com/site/about/guidelines.xhtml

22 Parikh R, Michelson D, Sapru M, *et al.* Priorities and preferences for school-based mental health services in India: a multi-stakeholder study with adolescents, parents, school staff, and mental health providers. *Glob Ment Health (Camb)* 2019;**6**. doi:10.1017/gmh.2019.16

23 Health promoting schools. https://www.who.int/health-topics/health-promoting-schools#tab=tab_1 [(accessed 25 June 2021]).

24 Srikala B, Kishore KKV. Empowering adolescents with life skills education in schools – School mental health program: Does it work? *Indian J Psychiatry* 2010;**52**:344–9. doi:10.4103/0019-5545.74310

25 Kumar D, Bharath S, Hirisave U, *et al.* School mental health programs in India: Current status and future directions. In: Weist MD, Kutcher S, Wei Y, eds. *School Mental Health: Global Challenges and Opportunities*. Cambridge University Press 2015: 95–104. doi:10.1017/CBO9781107284241.009

26 Shinde S, Weiss HA, Varghese B, *et al.* Promoting school climate and health outcomes with the SEHER multi-component secondary school intervention in Bihar, India: a cluster-randomised controlled trial. *Lancet* 2018;**392**:2465–77. doi:10.1016/S0140-6736(18)31615-5

27 Gujar K, Pingale V. School mental health programme in Maharashtra - Today and way ahead. *Ann Indian Psychiatry*;**1**:4. doi:10.4103/aip.aip_14_17

28 The State of the World's Children 2011: Adolescence - An age of opportunity. UNICEF DATA, 2011. Available: https://data.unicef.org/resources/the-state-of-the-worlds-children-2011-adolescents-an-age-of-opportunity/ [Accessed 8 May 2021].

29 Roy K, Shinde S, Sarkar BK, *et al.* India's response to adolescent mental health: a policy review and stakeholder analysis. *Soc Psychiatr Epidemiol* 2019;**54**:405–14. doi:10.1007/s00127-018-1647-2

30 Werner-Seidler A, Perry Y, Calear AL, *et al.* School-based depression and anxiety prevention programs for young people: A systematic review and meta-analysis. *Clin Psychol Rev* 2017;**51**:30–47. doi:10.1016/j.cpr.2016.10.005

31 Fazel M, Hoagwood K, Stephan S, *et al.* Mental health interventions in schools 1. *Lancet Psychiatry* 2014;**1**:377–87. doi:10.1016/S2215-0366(14)70312-8

32 Rajaraman D, Travasso S, Chatterjee A, *et al.* The acceptability, feasibility and impact of a lay health counsellor delivered health promoting schools programme in India: a case study evaluation. *BMC Health Serv Res* 2012;**12**:127. doi:10.1186/1472-6963-12-127

33 Shinde S, Weiss HA, Khandeparkar P, *et al.* A multicomponent secondary school health promotion intervention and adolescent health: An extension of the SEHER cluster randomised controlled trial in Bihar, India. *PLoS Med* 2020;**17**:e1003021. doi:10.1371/journal.pmed.1003021

34 Craig P, Dieppe P, Macintyre S, *et al.* Developing and evaluating complex interventions: the new Medical Research Council guidance. *BMJ* 2008;**337**:a1655. doi:10.1136/bmj.a1655

35 Wight D, Wimbush E, Jepson R, *et al.* Six steps in quality intervention development (6SQuID). *J Epidemiol Community Health* 2016;**70**:520–5. doi:10.1136/jech-2015-205952

For peer review only - http://bmjopen.bmj.com/site/about/guidelines.xhtml

36 World Health Organization. (2007). *Task shifting to tackle health worker shortages.* Geneva: WHO.

37 Movsisyan, A., Arnold, L., Evans, R. et al. Adapting evidence-informed complex population health interventions for new contexts: a systematic review of guidance. *Implement Sci* 2019; **14**:105. https://doi.org/10.1186/s13012-019-0956-5

38 Perera, C., Salamanca-Sanabria, A., Caballero-Bernal, J. et al. No implementation without cultural adaptation: a process for culturally adapting low-intensity psychological interventions in humanitarian settings. *Confl Health* 2020;**14**: 46. https://doi.org/10.1186/s13031-020-00290-0

39 Hugh-Jones S, Prabhu S, Lakshman KA, *et al.* The effectiveness of interventions to improve secondary school teachers' mental health literacy: a protocol for an umbrella review CRD42021250458. Published Online First: 2021. http://www.crd.york.ac.uk/PROSPERO/display_record.asp?ID=CRD42021250458

40 Hugh-Jones S, Prabhu S, Warriner L, *et al.* The effectiveness of universal school interventions for prevention or treatment of adolescent anxiety and depression: a protocol for an umbrella review. CRD42021248592. Published Online First: 2021. http://www.crd.york.ac.uk/PROSPERO/display_record.asp?ID=CRD42021248592

41 Hugh-Jones S, Prabhu S, Podiya JK, *et al.* The effectiveness of interventions to improve school climate in secondary schools: a protocol for an umbrella review. CRD42021251790. Published Online First: 2021. http://www.crd.york.ac.uk/PROSPERO/display_record.asp?ID=CRD42021251790

42 Hugh-Jones S, Prabhu S, Nag R, *et al.* The effectiveness of interventions to improve parent mental health literacy: a protocol for an umbrella review. CRD42021252006. Published Online First: 2021. http://www.crd.york.ac.uk/PROSPERO/display_record.asp?ID=CRD42021252006

43 Boustani M, Daleiden E, Bernstein A, *et al.* Using relevance mapping methodology to design an adolescent mental health intervention in India. *Glob Health Action* 2020; **13**:1. doi: 10.1080/16549716.2020.1775062

44 Hudson KG, Lawton R, Hugh-Jones S. (2020). Factors affecting the implementation of a whole school mindfulness program: a qualitative study using the consolidated framework for implementation research. *BMC Health Serv Res* 2020;**20**: 1-13. https://doi.org/10.1186/s12913-020-4942-z

45 Damschroder LJ, Aron DC, Keith RE, *et al.* Fostering implementation of health services research findings into practice: a consolidated framework for advancing implementation science. *Implement Sci* 2009;**4**:50. doi:10.1186/1748-5908-4-50

46 Stirman, S.W., Miller, C.J., Toder, K. et al. Development of a framework and coding system for modifications and adaptations of evidence-based interventions. *Implement Sci* 2013; **8**: 65). https://doi.org/10.1186/1748-5908-8-65

47 Bernal G, Sáez-Santiago E. Culturally centered psychosocial interventions. *J Community Psychol* 2006;**34:**121–32. doi:https://doi.org/10.1002/jcop.20096

48 Stirman, SW, Baumann AA, Miller, CJ. The FRAME: an expanded framework for reporting adaptations and modifications to evidence-based interventions. *Implement Sci2019;* ***14***(1), 1-10.

For peer review only - http://bmjopen.bmj.com/site/about/guidelines.xhtml

49 Lancaster GA, Dodd S, Williamson PR. Design and analysis of pilot studies: recommendations for good practice. *Jour Eval Clin Practice* 2004;10:307-312

50 Goodman R. Psychometric Properties of the Strengths and Difficulties Questionnaire. *J Am Acad Child Adolesc Psychiatry* 2001;**40**:1337–45. doi:10.1097/00004583-200111000-00015

51 Chorpita BF, Moffitt CE, Gray J. Psychometric properties of the Revised Child Anxiety and Depression Scale in a clinical sample. *Behav Res Ther* 2005;**43**:309–22. doi:10.1016/j.brat.2004.02.004

52 Wolpert M, Cheng H, Deighton J. Measurement Issues: Review of four patient reported outcome measures: SDQ, RCADS, C/ORS and GBO - their strengths and limitations for clinical use and service evaluation. *Child Adolesc Ment Health* 2015;**20**:63–70. doi:10.1111/camh.12065

53 Hugh-Jones S, Pert K, Kendal S*, et al.* MindMate2U: Development and feasibility study of a co-designed school-based mHealth intervention for symptomatic adolescents. JMIR Preprints. 22/04/2021:29834

54 Berry V, Axford N, Blower S, *et al.* The Effectiveness and Micro-costing Analysis of a Universal, School-Based, Social–Emotional Learning Programme in the UK: A Cluster-Randomised Controlled Trial. *School Ment Health* 2016;**8**:238–56. doi:10.1007/s12310-015-9160-1

55 Brazier J, Ratcliffe J, Saloman J, *et al. Measuring and Valuing Health Benefits for Economic Evaluation*. Oxford University Press 2017.

56 Stevens K. Developing a descriptive system for a new preference-based measure of health-related quality of life for children. *Qual Life Res* 2009;**18**:1105–13. doi:10.1007/s11136-009-9524-9

57 Ravens-Sieberer U, Wille N, Badia X, *et al.* Feasibility, reliability, and validity of the EQ-5D-Y: results from a multinational study. *Qual Life Res* 2010;**19**:887–97. doi:10.1007/s11136-010-9649-x

58 Herdman M, Gudex C, Lloyd A, *et al.* Development and preliminary testing of the new five-level version of EQ-5D (EQ-5D-5L). *Qual Life Res* 2011;**20**:1727–36. doi:10.1007/s11136-011-9903-x

59 Al-Janabi H, Flynn TN, Coast J. Development of a self-report measure of capability wellbeing for adults: the ICECAP-A. *Qual Life Res* 2012;**21**:167–76. doi:10.1007/s11136-011-9927-2

60 Gale NK, Heath G, Cameron E, *et al*. Using the framework method for the analysis of qualitative data in multi-disciplinary health research. BMC Med Res Methodol 2013; **13**: 117 (2013). https://doi.org/10.1186/1471-2288-13-117

61 Terry G, Hayfield N, Clarke V, *et al.* Thematic analysis. *The Sage Handbook of Qualitative Research in Psychology* 2017; pp 17-37.

62 Coast J. Is economic evaluation in touch with society's health values? *BMJ* 2004;**329**:1233–6. doi:10.1136/bmj.329.7476.1233

For peer review only - http://bmjopen.bmj.com/site/about/guidelines.xhtml

63 Mirzoev T, Das M, Ebenso B, *et al.* Contextual influences on the role of evidence in health policy development: what can we learn from six policies in India and Nigeria? *Evid Policy 2017;* **13**(1):59-79.

64 Mirzoev T, Green A, Gerein N, *et al.* 2013. Role of evidence in maternal health policy processes in Vietnam, India and China: findings from the HEPVIC project. *Evid Policy* 2013; **9**(4):493-511.

65 Lavis J, Oxman A, Lewin S, *et al.* SUPPORT Tools for evidence-informed health Policymaking (STP). *Health Res Policy Syst 2009;*7(Suppl 1):I1.

66 Green A, Gadsby E. 2007. A framework for evidence-informed health policy-making. In: *Green A, Bennett S, editors. Sound Choices: Enhancing Capacity for Evidence-Informed Health Policy AHPSR Biennual Review*. WHO, Alliance for Health Policy and Systems Research.

67 Global Consensus Statement, Meaningful Adolescent & Youth Engagement, 2018. Available: https://www.who.int/pmnch/mye-statement.pdf?ua=1 [Accessed 25 June 2021].

For peer review only

For peer review only - http://bmjopen.bmj.com/site/about/guidelines.xhtml

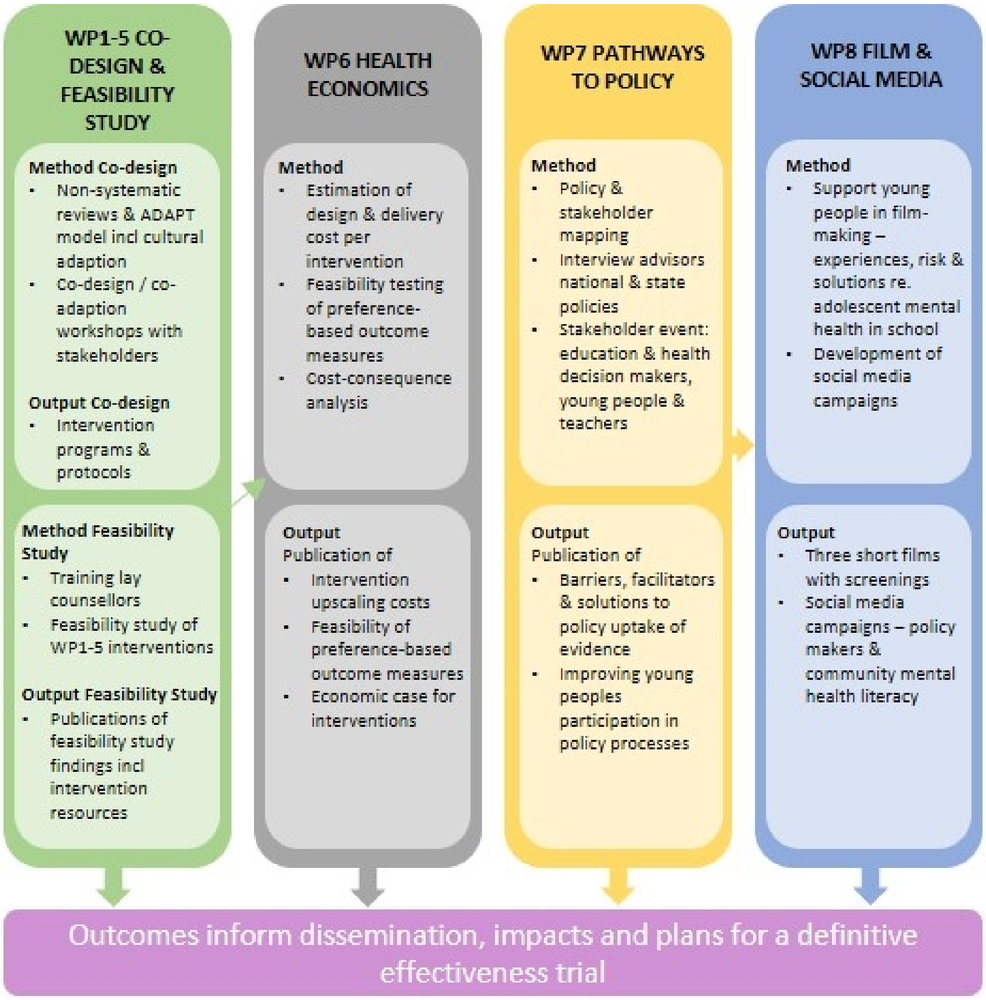

Fig 1 Study work packages

89x90mm (150 x 150 DPI)

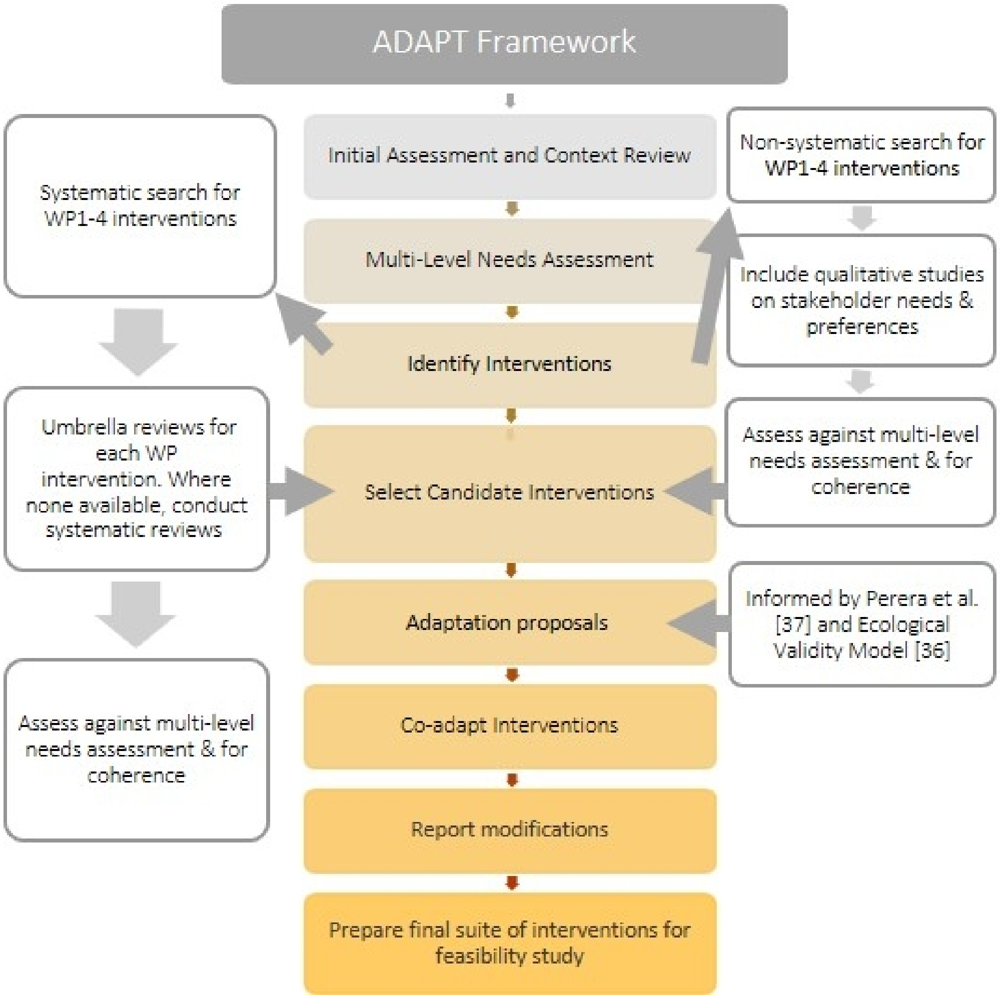

Figure 2 Stages of co-adaptation

90x90mm (150 x 150 DPI)
